# Social Context Matters for Turn‐Taking Dynamics: A Comparative Study of Autistic and Typically Developing Children

**DOI:** 10.1111/cogs.70124

**Published:** 2025-10-13

**Authors:** Christopher Cox, Riccardo Fusaroli, Yngwie A. Nielsen, Sunghye Cho, Roberta Rocca, Arndis Simonsen, Azia Knox, Meg Lyons, Mark Liberman, Christopher Cieri, Sarah Schillinger, Amanda L. Lee, Aili Hauptmann, Kimberly Tena, Christopher Chatham, Judith S. Miller, Juhi Pandey, Alison S. Russell, Robert T. Schultz, Julia Parish‐Morris

**Affiliations:** ^1^ Department of Linguistics, Cognitive Science and Semiotics Aarhus University; ^2^ Interacting Minds Center Aarhus University; ^3^ Linguistic Data Consortium University of Pennsylvania; ^4^ Department of Linguistics University of Pennsylvania; ^5^ Psychosis Research Unit, Psychiatry Aarhus University Hospital; ^6^ Department of Linguistics and Cognitive Science University of Delaware; ^7^ Center for Autism Research Children's Hospital of Philadelphia; ^8^ Carolina Autism and Neurodevelopment Research Center University of South Carolina; ^9^ Hoffmann‐La Roche Ltd; ^10^ Department of Psychiatry Perelman School of Medicine at the University of Pennsylvania; ^11^ Department of Child and Adolescent Psychiatry and Behavioral Sciences Children's Hospital of Philadelphia; ^12^ Department of Psychology and Neuroscience University of North Carolina Chapel Hill

**Keywords:** Turn‐taking, Autism spectrum disorder, Conversational dynamics, Social context, Child development, Interpersonal coordination, Response latency, Social cognition

## Abstract

Engaging in fluent conversation is a surprisingly complex task that requires interlocutors to promptly respond to each other in a way that is appropriate to the social context. In this study, we disentangled different dimensions of turn‐taking by investigating how the dynamics of child–adult interactions changed according to the activity (task‐oriented vs. freer conversation) and the familiarity of the interlocutor (familiar vs. unfamiliar). Twenty‐eight autistic children (16 male; $M_{age}$ = 10.8 years) and 20 age‐matched typically developing children (8 male; $M_{age}$ = 9.6 years) participated in seven task‐orientated face‐to‐face conversations with their caregivers (336 total conversations) and seven more telephone conversations alternately with their caregivers (144 total conversations, 60 with the typical development group) and an experimenter (191 total conversations, 112 with the autism group). By modeling inter‐turn response latencies in multi‐level Bayesian location‐scale models, we found that inter‐turn response latencies were consistent across repeated measures within social contexts, but exhibited substantial differences across social contexts. Autistic children exhibited more overlaps, produced faster response latencies and shorter pauses than typically developing children—and these group differences were stronger when conversing with the unfamiliar experimenter. Unfamiliarity also made the relation between individual differences and latencies evident: only in conversations with the experimenter were higher sociocognitive skills and lower social awareness associated with faster responses. Information flow and shared tempo were also influenced by familiarity: children adapted their response latencies to the predictability and tempo of their interlocutor's turn, but only when interacting with their caregivers and not the experimenter. These results highlight the need to construe turn‐taking as a multicomponential construct that is shaped by individual differences, interpersonal dynamics, and the affordances of the context.

## 1 Introduction

Humans navigate their social world through conversations. Despite their apparent ease and naturalness, fluent conversations are complex feats of coordination involving rapid responses that operate at the absolute limits of human reaction times. The typical gap between conversational turns lies at approximately 59–200 ms (Dingemanse & Liesenfeld, 2022; Levinson, 2016; Stivers et al., 2009), which is too fast to rely on reactive planning and production after the end of the previous turn (Magyari, Bastiaansen, De Ruiter, & Levinson, 2014; Magyari & De Ruiter, 2012). Interlocutors must, therefore, proactively prepare their responses, predict the current turn end, and engage their articulators ahead of time (Dale, Fusaroli, Duran, & Richardson, 2013; Magyari & De Ruiter, 2012; Magyari et al., 2014). While adults draw upon their linguistic, motor, and interpersonal skills as well as world knowledge to plan and predict with such efficiency (Ford, Fox, & Thompson, 1996; Magyari & De Ruiter, 2012; Magyari et al., 2014), it remains an open question how children manage this complex act of coordination. In fact, response latency has been taken as a simple (one‐to‐one) proxy of social competence without regard for the contextual and intraindividual processes that underlie turn‐taking. In this paper, we investigate how child–adult turn‐taking dynamics are influenced by the social context, the moment‐to‐moment dynamics of the conversation, and the individual differences in linguistic, sociocognitive, and motor skills of both typically and atypically developing children.

### 1.1 Turn‐taking as social competence

Turn‐taking emerges early in ontogeny (Levinson, 2016), is exhibited by our nearest phylogenetic ancestors and across the animal kingdom (Cox, Templeton, & Fusaroli, 2023; Levinson, 2006, 2016; Okobi Jr, Banerjee, Matheson, Phelps, & Long, 2019; Ravignani & de Reus, 2019; Verga, Kotz, & Ravignani, 2023), and displays similar structures across languages (Dingemanse & Liesenfeld, 2022; Stivers et al., 2009). To explain this ubiquity, turn‐taking has been argued to arise from a “package” of fundamental capacities enabling human social behavior (Levinson, 2006, 2016). Accordingly, turn‐taking has important social implications; shorter inter‐turn latencies facilitate efficient information exchange (Fusaroli, Rączaszek‐Leonardi, & Tylén, 2014; Templeton, Chang, Reynolds, Cone LeBeaumont, & Wheatley, 2022, 2023) and foster a sense of connection and shared motivation (Dideriksen, Christiansen, Tylén, Dingemanse, & Fusaroli, 2023; Fusaroli, Weed, Fein, & Naigles, 2019; Fusaroli & Tylén, 2016; Konvalinka & Roepstorff, 2012; Tylén, Fusaroli, Bundgaard, & Østergaard, 2013). Adults in verbal interactions with shorter inter‐turn response latencies, for example, report enjoying their conversations more (Templeton et al., 2022).

In light of these findings, there is a broad tendency to equate short turn‐taking latencies with skillful turn‐taking, and in turn, to equate individual variation in turn‐taking latency to the expression of social competence (Cho et al., 2023; Fusaroli et al., 2025). However, the shortcomings of this perspective are increasingly becoming apparent, especially within developmental and clinical work. Several empirical studies have documented slower turn‐taking latencies in children with autism (Fay & Schuler, 1980; Heeman, Lunsford, Selfridge, Black, & Van Santen, 2010; Ochi et al., 2019; Warlaumont, Richards, Gilkerson, & Oller, 2014), leading to the mainstream conception that autistic children are slower due to a social competence deficit (see Nguyen, Versyp, Cox, & Fusaroli, 2022 for a systematic review). However, recent studies have either failed to replicate this finding (Cho et al., 2023; Wehrle, Cangemi, Janz, Vogeley, & Grice, 2023) or obtained the opposite pattern, namely, faster turn‐taking in autistic children (Fusaroli et al., 2025). Part of the explanation lies in the observation that children with autism exhibit both larger overlaps (i.e., instances of starting to speak before the previous speaker is done) and longer‐tailed distributions (i.e., instances of very long gaps), suggesting that autistic children may initiate their turns faster or slower than typically developing children, depending on the context (Fusaroli et al., 2025). These findings dovetail with qualitative investigations showing that autistic children generally do not display difficulties in conversational turn‐taking with caregivers (Bottema‐Beutel, Lloyd, Watson, & Yoder, 2018; Ochs, Kremer‐Sadlik, Sirota, & Solomon, 2004), possibly due to caregivers scaffolding turn‐taking by producing utterances that invite contingent responses (Bottema‐Beutel et al., 2018; Bottema‐Beutel, Crowley, & Kim, 2022; see also Dunn & Shatz, 1989; Ervin‐Tripp, 1982 for evidence in typically developing children). Further, developmental trajectories can be very different from child to child, and this heterogeneity is even more prominent in autism (Fusaroli et al., 2019). Contrasting autistic and typically developing children as groups risks neglecting more proximal causes of differences in turn‐taking behaviors, such as crucial variation in linguistic, sociocognitive, and motor abilities, and how they play out in different contexts. These findings suggest the need to look beyond turn‐taking as a monolithic construct, focusing instead on how turn‐taking emerges from the interplay of individual differences, interpersonal dynamics, and contextual demands.

### 1.2 Unfolding the components of turn‐taking

No two conversations are the same. A relaxed chat and an oral examination posit wildly different contextual demands on the conversation, including the complexity of the linguistic content to plan and process as well as what is considered an appropriate turn‐taking latency. Likewise, no two individuals are the same, and turn‐taking dynamics likely depend on several linguistic, sociocognitive, and motor skills. In the following, we construe the temporal dynamics of turn‐taking as a window into this complex network of interacting factors and argue that a nuanced perspective on verbal interactions requires consideration of the following components: the sociocognitive, linguistic and motor skills of the speaker, the properties of the individual utterances, and the unfolding interpersonal processes of the speakers. How these individual components play out in turn‐taking depends on contextual demands, including the purpose of the conversation and the relation between interlocutors.

#### 1.2.1 Purpose of the conversation

Verbal interactions do not happen in a vacuum, but emerge as an intrinsic part of joint activities with distinct affordances and constraints (Cola et al., 2022; Dale, 2015; Fusaroli et al., 2014; Fusaroli & Tylén, 2012; Fusaroli, Gangopadhyay, & Tylén, 2014; Olsen & Tylén, 2023; Parish‐Morris et al., 2019). These affordances and constraints may directly affect turn‐taking dynamics. For instance, a joint task that requires children and parents to tell each other where to place stickers on a blank picture demands a focus on precise information transfer. More importantly, it requires interlocutors to allow each other sufficient time to place the stickers before responding. In contrast, the situational demands of a spontaneous conversation about holidays demand less precision, presumably resulting in fewer long pauses (see Dideriksen et al., 2023; Dideriksen et al., 2023; Dideriksen, Fusaroli, Tylén, Dingemanse, & Christiansen, 2019; Fusaroli et al., 2017 for examples of asymmetric access to knowledge, or asymmetric roles).

#### 1.2.2 Relation to the interlocutor

Equally important is the person we interact with in joint activities. Different interlocutors share different levels of common ground (i.e., assumed common knowledge; (Branigan, Bell, & McLean, 2016; Clark, 1996; De Marchena & Eigsti, 2016)), which provides the conditions for transferring information. For instance, chatting with a long‐known friend is very different from talking with a stranger at a party, and discussing which toy to bring at a show‐and‐tell at school is very different if doing so with a peer compared to a teacher. In child–adult interactions, the familiarity of the interlocutor changes how both autistic and typically developing children interact (Doherty‐Sneddon, Whittle, & Riby, 2013; Dawson et al., 2002; Forgeot d'Arc, Devaine, & Daunizeau, 2020). Neurotypical adults interacting with friends likewise take longer to respond compared to when interacting with strangers, and their occasional long pauses are perceived more positively (Templeton et al., 2022, 2023).

Turn‐taking patterns in child–adult conversations, then, represent an interaction between the structural demands of the task and the degree of familiarity with an interlocutor. The study design of the current paper allowed us to evaluate how different affordances emerging from the task and the familiarity of the interlocutor produced differences between the autism and typical development group in their turn‐taking behaviors (see Research Questions 1 & 2 below). Children and adults were recorded in each of these social contexts in multiple weekly sessions to disentangle heterogeneity due to session‐by‐session variation from systematic contextual variation.

#### 1.2.3 Individual differences

The challenges posed by various contextual demands and interlocutors require a complex network of skills to overcome. For instance, promptly responding requires mature linguistic skills; that is, the ability to integrate semantic, syntactic, and pragmatic information to anticipate an imminent termination of a turn (Magyari & De Ruiter, 2012). According to the *Interaction Engine Hypothesis*, human sociocognitive skills lie at the core of turn‐taking (Levinson, 2016), suggesting a role for individual differences in social skills. Thus, smooth turn‐taking also requires the speaker to infer others' intentions, anticipating what they might do next, and plan behaviors that are predictable for the interlocutor (Heesen & Fröhlich, 2022; Levinson, 2006; Waade, Enevoldsen, Vermillet, Simonsen, & Fusaroli, 2023). In addition, motor development may be important as it affects the nature of children's interactions with the world around them, making them better at promptly anticipating, orienting toward, and reacting to information sources (Iverson, 2010). These skills have crucial downstream effects on their own communicative abilities (West & Iverson, 2021), as well as on the contingent responses from caregivers (Karasik, Tamis‐LeMonda,& Adolph, 2014; Tamis‐LeMonda & Adolph, 2013). Besides directly affecting the children's behaviors, individual differences in skills change the input that children receive from caregivers; several studies of adults have shown that they adapt to their addressees, including non‐native speakers (Piazza, Martin, & Kalashnikova, 2022; Uther, Knoll, & Burnham, 2007), autistic and nonautistic children (Cox et al., 2023; Hilton et al., 2022; Leezenbaum, Campbell, Butler, & Iverson, 2014; Neimy, Pelaez, Carrow, Monlux, & Tarbox, 2017; Warlaumont et al., 2014), infants with cochlear implants (Dilley et al., 2020; Kondaurova, Bergeson, & Xu, 2013), or pets (Jardat et al., 2022; Panneton, Cristia, Taylor, & Christine, 2023).

Crucially, different contextual and interlocutor demands might engage these individual skills to a higher or lower degree. For instance, the behavior of familiar interlocutors might be easier for children to anticipate and respond to, while the ability to promptly take turns with an unfamiliar interlocutor might be more dependent on sociocognitive skills (Bigelow, 1999; Jaffe et al., 2001). In other words, assessing how individual differences relate to response latencies as we vary the characteristics of the social context is essential to improving our understanding of their role in turn‐taking. To assess these ideas, our study incorporated rich measures of individual differences to examine how sociocognitive, linguistic, and motor differences among children might change the turn‐taking dynamics of interactions across social contexts with different task demands (see Research Question 3 below).

#### 1.2.4 Properties of the utterance

Conversations are dynamic, and conversational demands vary on a turn‐by‐turn basis in terms of the novelty of information, the effort required to parse what has been said, and the effort required to plan the next utterance. In adults, inter‐turn response latencies depend on the predictability of the content discussed (Bögels, Magyari, & Levinson, 2015), and preschool children produce shorter inter‐turn response latencies with more predictable ends to questions (Casillas & Frank, 2017; Lindsay, Gambi, & Rabagliati, 2019). Intuitively, children having to respond to a complex, open‐ended question (e.g., “What are your thoughts on the story we just read?”) will likely produce longer response times compared to a predictable, closed‐ended question (e.g., “Do you have an apple in your hand?”).

To assess this component, our study quantified two distinct aspects of predictability. First, we measured the processing effort required for an incoming utterance by calculating how predictable it was from previous turns (to investigate whether more predictable utterances require less processing effort, potentially enabling faster responses). Second, we measured the planning effort for an outgoing utterance by calculating how predictable the speaker's upcoming utterance was compared to previous turns. For this planning aspect, we investigated whether more predictable utterances might actually require more or less coordination with the ongoing conversation flow. On the one hand, highly predictable responses might demand closer attention to the conversational context and more careful coordination, possibly resulting in longer planning time. On the other hand, highly predictable responses might build on previous utterances and, therefore, require less effort and cognitive load. We then assessed whether these complementary measures of predictability had reliable associations with inter‐turn response latencies (see Research Question 4 below).

#### 1.2.5 Interpersonal dynamics

Beyond pure exchanges of information, conversations are also a social dance in which interlocutors might come to share a common tempo of well‐timed exchanges (Fusaroli et al., 2014; Wilson & Wilson, 2005). For example, in a conversation between a child and caregiver during a routine activity (e.g., preparing a meal), the child might naturally match the caregiver's rhythm, responding with pauses that mirror the caregiver's slower pace. However, during a more structured activity, like a reading lesson, the caregiver might adopt a faster tempo, prompting the child to respond more quickly. This shift in tempo is not just a matter of individual response speed but reflects an ongoing process of adaptation, where both participants adjust their timing in response to the conversational and social context, as well as to each other (Pouw & Holler, 2022; Zamm, Pfordresher, & Palmer, 2015). There are general findings of strong correlations between child and caregiver response latencies (Nguyen et al., 2022); however, these findings are based only on summary estimates (the average response latency across the whole conversation) and cannot say much about the dynamics of co‐adaptation on a turn‐by‐turn basis, nor how context demands or individual differences influence such co‐adaptation (Fusaroli et al., 2025). Further, vocal interactions likely involve dynamic ebb‐and‐flow waves of mutual convergence and scaffolding that takes place during local transactions (Pouw & Holler, 2022; Ritwika et al., 2020; Warlaumont et al., 2014), but little is known about how this unfolds in child–adult interactions. Accordingly, our study included an analysis of these temporal dependencies: how child and caregiver adjust to each other's—and their own—previous latencies on a turn‐by‐turn basis across contexts (see Research Question 5 below).

### 1.3 The present research

In the present research, we unpack different components of turn‐taking and investigate how they unfold across diverse social contexts, in autism and typical development; and unravel contradictory results pertaining to turn‐taking in autistic children. Specifically, we focus on the following five research questions (RQs):

- **RQ1**: Are response latencies an individual trait, or do differences between sessions and social contexts (conversational task and familiarity of the interlocutor) matter?
- **RQ2**: Does autism on average involve slower response latencies than typical development—once explicitly accounting for long pauses and overlaps? How is the finding shaped by context?
- **RQ3**: Are differences in turn‐taking behavior grounded in individual differences (social cognition, social awareness and social motivation, language skills and motor skills)? How are the findings shaped by context?
- **RQ4**: Is turn‐taking behavior across social contexts grounded in the turn‐by‐turn information flow (predictability of previous and current utterance)?
- **RQ5**: Is turn‐taking behavior across social contexts grounded in the turn‐by‐turn temporal adjustments in the interaction?

We study these RQs using a corpus of child–adult conversations with both neurotypical and autistic children, several contexts (i.e., free and task‐orientated conversations as well as familiar and unfamiliar interlocutors), measures of individual differences (i.e., social, cognitive, motor skills), information flow (i.e., utterance predictability), and turn‐by‐turn temporal adjustments (i.e., self and interpersonal adjustment).

## 2 Methods

### 2.1 Participants

The study involved 28 autistic (10 female, 2 nonbinary) and 20 typically developing children (12 female), matched at the group level on age, IQ, and self‐reported race (see Table 1). All autistic participants had previously received a clinical diagnosis of autism spectrum disorder (ASD) from licensed psychologists or physicians external to the research center. When available, previous documentation of diagnostic reports, previous evaluations, and Individualized Education Program documentation was requested. While some diagnostic documentation may have been based on earlier frameworks (i.e., DSM‐IV), the confirmatory evaluation procedures aligned with DSM‐5 criteria. Such diagnostic confirmation at the research center included the Autism Spectrum Disorder Diagnostic Interview and Activities‐Lifespan (ASD‐DIAL), the Childhood Autism Rating Scale, 2nd Edition (CARS‐2), standardized cognitive and language assessments, and parent‐reported questionnaires (e.g., SCQ, SRS‐2) to confirm ASD diagnosis or lack thereof. Participants were recruited via an electronic health record system at a large hospital‐based academic medical center in the United States, flyers and inquiry forms, and internal databases. All participants underwent the Telephone Screening Interview (TSI) developed by the researchers at the Center for Autism Research to ascertain adherence to inclusion and exclusion criteria (such as autism diagnostic history, family history, or upcoming changes to intervention/medication). The inclusion criteria were as follows: all participants had to (i) be between 6 and 18 years of age; (ii) have English as their primary language; (iii) be verbally fluent consistent with their chronological age; (iv) have an IQ score above 75; and (v) have a current Social and Communication Questionnaire (SCQ) score above 10 (for autistic children) or below 11 (for neurotypical children), where higher scores represent a higher degree of social communication impairment (Berument, Rutter, Lord, Pickles, & Bailey, 1999; Chesnut, Wei, Barnard‐Brak, & Richman, 2017). Verbal fluency was assessed through standardized language evaluations and clinical judgment to ensure all participants could engage meaningfully in conversational tasks. The exclusion criteria were as follows: all participants had (i) no known genetic or neurological condition that impacts neurodevelopment, language, or vocal production; (ii) no history of expressive language deficits or severe neurological injury likely to affect expressive language and communication behavior; (iii) no extreme birth prematurity (<32 weeks); (iv) no diagnosed hearing impairment or cochlear implant; (v) no plan to begin or change medication or intervention during study duration; and (vi) no first‐degree family members with autism (for the neurotypical children only).

**Table 1:** Descriptive statistics of the sample

Variable	Autism Group	Typical Development
Gender	10 female, 16 male, 2 nonbinary	12 female, 8 male
Age (years)	10.82 (3.14)	9.6 (3.02)
Age Range (years)	6–17	6–15
IQ	112.88 (13.26)	118.16 (13.15)
SCQ	16.68 (16.68)	1.20 (1.20)
SRS (Raw)	77.57 (25.99)	12.75 (9.13)
SRS (T‐score)	67.77 (9.92)	42.50 (3.44)
Language (CELF‐5)	107.67 (13.15)	109.53 (11.40)
Social Cognition (SRS‐2)	13.71 (5.46)	1.8 (2.21)
Social Motivation (SRS‐2)	12.36 (6.11)	3 (2.13)
Social Awareness (SRS‐2)	10.39 (3.51)	2.5 (2.04)
Motor (VAB‐3)	82.54 (3.82)	85.25 (2.07)
Number of Children	28	20
Number of Sessions	392	279
Number of Turns	21733	14628
Average Turns Per Session	27.7	26.3
Median Turn Length (s)	2.87 (8.22)	2.69 (9.93)
Mean Turn Length (s)	5.69 (8.22)	5.97 (9.93)

*Note*. Numbers in parentheses denote standard deviations for each of the variables. The last five rows display details about the turns for each of the groups under investigation. Median and Mean Turn Length refer to the average difference in seconds between the startpoint and endpoint of children's turns across conditions. Abbreviations: CELF‐5, Clinical Evaluation of Language Fundamentals‐5th edition; IQ, Intelligence Quotient, as measured with the Wechsler Abbreviated Scales of Intelligence‐2nd Edition; SCQ, Social and Communication Questionnaire; SRS‐2, Social Responsiveness Scales‐2nd Edition; VAB‐3, Vineland Adaptive Behavior‐3rd edition.

### 2.2 Procedure

#### 2.2.1 Data collection

Participant data were collected over the course of several telephone sessions. After an information call and having provided consent, the participants and their families underwent the screening questionnaire (TSI) and the Social and Communication Questionnaire (Berument et al., 1999; Chesnut et al., 2017). Subsequent assessment relied on the Wechsler Abbreviated Scale of Intelligence, 2nd Edition (WASI‐II) (McCrimmon & Smith, 2013; Wechsler, 2008), the Clinical Evaluation of Language Fundamentals, 5th Edition (CELF‐5) (Denman et al., 2017; Wiig, Secord, & Semel, 2013), the Vineland Adaptive Behavior Scales, 3rd Edition (Vineland‐3) (Farmer, Adedipe, Bal, Chlebowski, & Thurm, 2020; Sparrow, Cicchetti, & Saulnier, 2016), and the Social Responsiveness Scale (SRS‐2), 2nd edition (Bruni, 2014; Constantino, 2012). While the conversational data were collected via telephone, all standardized assessments (WASI‐II, CELF‐5, Vineland‐3, and SRS‐2) were administered during in‐person research visits by trained clinicians following standard administration protocols.

After evaluation, according to the above assessment tools, participants underwent phone‐based recording sessions approximately once per week for 7 weeks. The participants participated in two speech recording tasks, namely, a matching game (only performed with the parent) and a prompted conversation (alternately with the parent and with an unfamiliar research assistant). These two tasks were adapted from established paradigms in conversational research (Dideriksen et al., 2023; Fay et al., 2018) to be engaging and repeatable for our age range (6–17 years). In the matching game, the child and parent each received a sticker sheet and a blank picture and were instructed to take turns in telling the other person where to place the stickers. The aim of the game was to obtain identical pictures despite not being able to see each other's sticker sheet. In the prompted conversations, children were given guided prompts (e.g., birthday party planning, hobbies, food preferences) and interacted alternately with their caregivers in even weeks and with an initially unfamiliar experimenter in odd weeks. Participants were asked to chat for approximately 5 min, though actual durations varied naturally (see Table 2). The matching game with parents typically lasted longest, while parent conversations were slightly briefer than those with experimenters.

**Table 2:** Average task duration (in min)

Task	Autism Group	Typical Development
Matching With Parent	18.7 (7.44)	17.3 (6.45)
Convo With Parent	3.52 (1.62)	3.50 (1.48)
Convo With Experimenter	5.25 (3.14)	4.89 (2.02)

*Note*. Values represent mean durations in minutes with standard deviations in parentheses.

All recording sessions occurred in participants' homes with caregivers present to assist children in conversations with the experimenter when needed. During the first session with the child, the research assistant remained on the line to facilitate tasks with the parent and child, while in following sessions, children and parents completed the tasks on their own. We aimed to maintain consistent research assistant assignments for each participant across all sessions, but this was not always feasible due to scheduling constraints and research assistant availability (see Fig. S22 for an overview of research assistant assignments for each participant and Fig. S23 for a control analysis). Phone calls were scheduled based on family availability, and we tried to maintain consistency in session times where possible. The sequence of tasks was not counterbalanced across visits or participants; all sessions followed the same standardized protocol where participants first completed the matching game followed by the prompted conversation. This consistent ordering was maintained across all seven weekly visits and all participants to ensure procedural consistency. All technical issues were systematically documented, with detailed session‐by‐session breakdowns provided in Table S15. Regarding the sequence of conversations, participants spoke with unfamiliar partners (research assistants) during odd‐numbered weeks and caregivers during even‐numbered weeks, completing both activities (matching game and conversation) within the same phone call when speaking with caregivers. This experimental setup implies that we have the following number of sessions for each child: seven sessions for the matching game with parents, four sessions of conversations with the experimenter, and three sessions of conversations with the parent.

#### 2.2.2 Recording and transcribing turns

To record participants' speech, we relied on an automated phone bank collection system developed by the University of Pennsylvania Linguistic Data Consortium and adapted for use with autistic participants and across a wide variety of different devices. The speech recordings were transcribed by trained annotators using a web‐based transcription tool with a built‐in speech activity detector function. Data transcription involved annotators assessing and correcting the timestamps for automatically identified speech segment boundaries. Analyses of inter‐transcriber reliability of speech segment boundaries demonstrated excellent agreement (κ = 0.88 [0.74, 0.97] for conversations and κ = 0.88 [0.76, 0.94] for the matching game, with percentage agreement across all recordings was 94.7%, see Supplementary Materials). Conversational turns were conceptualized as an idealized ABABAB structure, where each speaker takes sequential turns. In practice, overlapping speech sometimes occurred, which we handled through specific segmentation procedures to preserve the sequential analysis framework. When both interlocutors produced speech simultaneously, we combined backchannels with the subsequent turn by the same interlocutor, calculating latency based on the timing of substantial content following the backchannel rather than the backchannel onset itself. This approach maintained the ABABAB structure while accounting for natural conversation dynamics. Utterances with negative latencies thus represent cases where a full utterance (not a backchannel) began before the previous speaker had completed their turn.

### 2.3 Operationalizing turn‐taking

Inter‐turn response latencies were calculated by subtracting the onset of an utterance from the offset of the previous utterance. This yielded both negative (overlaps) and positive response latencies (gaps), including a nontrivial number of long pauses. In previous research, overlaps have often been excluded as failures of communication, or have been considered to be qualitatively different from gaps (Fusaroli et al., 2025; Nguyen et al., 2022; Ochi et al., 2019). Long pauses have likewise either been included but ignored (i.e., using likelihood functions without long right tails), or been excluded as outliers at various thresholds changing from study to study (Clark & Lindsey, 2015; Gratier et al., 2015). These different conceptions of turn‐taking can lead to different findings even when using the same dataset. For example, two studies explicitly including overlaps found that response latencies for the autism group were equivalent or faster than those of the typical development group (Fusaroli et al., 2025; Wehrle et al., 2023), contrary to studies excluding overlaps (Nguyen et al., 2022; Ochi et al., 2019).

In this study, we chose to include overlaps and long pauses and to explicitly account for them in the statistical model. We made this choice because overlaps constitute a substantial portion of conversations: between 25% and 50% of conversational utterances are estimated to start before the previous interlocutor is done with speaking (Dingemanse & Liesenfeld, 2022), and this is argued to be even more frequent in conversations involving children (Ervin‐Tripp, 1979; Garvey & Berninger, 1981). Moreover, there is a growing recognition that overlaps may serve several different functions, similar to how long gaps may signify both thoughtful engagement of the interlocutor or a desire to avoid a topic (Templeton, Chang, Reynolds, Cone LeBeaumont, & Wheatley, 2023). Specifically, overlaps can indicate disengaging from the conversation and interruptions, but also shared attention and mutual understanding (as in backchannels), completion of the interlocutor's utterance, or they may signal disagreement and the need for potential repair (Dideriksen et al., 2023). Overlaps thereby play a crucial role in building and maintaining rapport (Bryant et al., 2016; Bryant, 2020; Cummins, 2019; Templeton et al., 2022). Together with long pauses, overlaps form a natural part of smooth turn‐taking, and, we argue, should be integrated into models of turn‐taking instead of being discarded. Beyond the conceptual reasons for including overlaps, removing overlaps and long pauses also truncates an otherwise continuous distribution, which violates traditional distributional assumptions (Fusaroli et al., 2025). To nonetheless allow for comparisons with prior studies (for a review see Nguyen et al. (2022) and highlight the impact of our modeling choices, we conducted additional analyses following the prior work (i.e., excluding overlaps, no explicit modeling of long pauses). We report these in the Supplementary Materials.

### 2.4 Statistical models

**Fig. 1:**
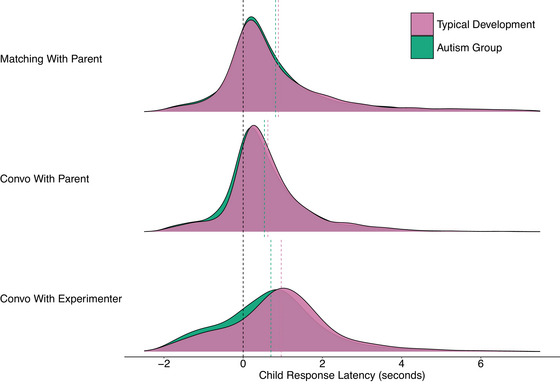
Plots to demonstrate the distributional properties of child response latencies across different combinations of contexts and familiarity. The black dashed line marks a response latency of zero, the purple dashed line marks the mean in the typical development group, and the green dashed line marks the mean of the autism group. As can be gleaned from the plots, response latencies involve both negative response latencies (overlapping speech), a large center of mass just above zero, as well as positive skew (long pauses between turns).

The distributional properties of response latencies in turn‐taking produce unique modeling challenges (see Fig. 1). Turn‐taking distributions involve negative latencies from overlapping speech, a large bulk of observations just above zero, and positive skew from occasional long pauses between turns (Fusaroli et al., 2025; Heldner & Edlund, 2010). To accommodate these distributional properties in our models, we applied Bayesian multilevel models with ex‐Gaussian likelihoods for all of our research questions. This form of likelihood combines a Gaussian distribution with an exponential distribution, allowing for the flexibility to represent the central tendency together with the positive skew often observed in empirical turn‐taking data. Specifically, in our Bayesian framework, the ex‐Gaussian likelihood was parameterized by three parameters: the mean (μ) and variance (σ) of the Gaussian component, as well as the rate (β) of the exponential tail. Control analyses using simpler distributional assumptions (see Supplementary Materials) demonstrated that failing to account for the exponential tail component would lead to biased estimates of central tendency, particularly in contexts with more frequent long pauses such as the matching game. The ex‐Gaussian explicitly models these distributional characteristics, allowing us to separately quantify differences in typical response timing from differences in the propensity for long pauses, thereby providing a more complete picture of turn‐taking dynamics across groups and contexts.

To investigate whether response latencies are an individual trait (RQ1) and whether autism on average involves slower response latencies than typical development (RQ2), we conditioned each of the parameters—the Gaussian mean (μ) and variance (σ), as well as the exponential rate (β)—on the diagnostic group (neurotypical vs. autistic children) and conversational context (matching game vs. conversations with parents vs. conversations with experimenters). To assess group differences across the tasks and familiarity of the interlocutor, we allowed the estimates to vary within each combination of these factors (similar to a full interaction model). To account for the hierarchical structure of our data, we implemented a multilevel Bayesian model with random effects at both participant and visit levels. At the participant level, random slopes were included for each Task‐Familiarity combination. At the visit level, random slopes captured how the effects of different Task‐Familiarity combinations varied across the seven weekly sessions. While not all combinations occurred in every visit (e.g., conversations with unfamiliar partners only occurred in odd‐numbered weeks), this structure accounted for visit‐specific effects that might influence participants' turn‐taking behaviors over time. Variance was partially pooled by child within their respective diagnostic group to accommodate the potential for between‐group differences in heterogeneity in mean and variance. To investigate the test‐retest reliability of our measures of response latency, we calculated correlations between individual participants' mean latencies across repeated visits within each social context (all seven visits for matching game; visits 2, 4, 6 for parent conversations; visits 1, 3, 5, 7 for experimenter conversations) with bootstrapped confidence intervals (*N* = 1000). We also assessed cross‐context consistency by correlating each child's mean latency estimates between the three social contexts to examine whether individual differences in response timing represent stable characteristics that persist across different social situations. More information about the specification of our models, including priors, model checks, and control analyses, can be found in Supplementary Materials.

To investigate the role of individual differences on turn timing (RQ3), we added scaled scores of SRS‐2 (Social Cognition, Social Motivation, Social Awareness), CELF‐5 (Language), and VAB‐3 (Motor) to the first model (see Table 1). The Vineland Adaptive Behavior Scales, Third Edition (VAB‐3) is standardized for individuals from birth through 90 years of age, with age‐appropriate norms that allow for meaningful comparisons across our participants despite age differences. We should note that while we used general motor skills from the Vineland Adaptive Behavior Scales (VAB‐3) to provide a broad assessment of motor abilities, a limitation of this approach is the absence of specific oral‐motor or speech‐motor measures, which might be more directly relevant to turn‐taking mechanisms given recent evidence linking speech‐motor networks to timing differences in autism (O'Brien et al., 2023). Rather than treating social skills as a unitary construct, moreover, we distinguished between three key dimensions from the SRS‐2 inventory: social cognition, social awareness, and social motivation (Bruni, 2014; Constantino, 2012). While we acknowledge that the scaled scores from three subscales of the SRS‐2 were originally designed for clinical reference rather than as independent research variables, they provide useful constructs for examining different aspects of social functioning. These distinct but related dimensions of social functioning may contribute differently to turn‐taking dynamics in conversation. Social awareness involves the ability to perceive social cues (e.g., noticing changes in facial expressions or shifts in tone of voice), while social cognition refers to the ability to interpret social cues (e.g., understanding that a person leaning forward indicates interest or that a brief silence might signal confusion). Social motivation captures the drive to engage in social interaction and form interpersonal bonds. These distinct but related dimensions of social functioning may contribute differently to turn‐taking dynamics in conversation. For example, social awareness might enhance detection of turn‐yielding cues, social cognition might facilitate anticipation of turn completions, and social motivation might influence the effort devoted to maintaining conversational timing. Their relation to response latencies was allowed to vary by diagnostic group, conversational context, and familiarity (equivalent to a full interaction model), enabling us to assess how these relationships differed across contexts. We chose to use SRS scores of sociocognitive skills instead of Vineland Socialization scores since individual abilities were measured only once during data collection. Vineland scores are argued to be more sensitive to small variations between weekly sessions than SRS scores and, therefore, more likely to introduce noise in the association between underlying social skills measured at one point and response latencies measured over several sessions (Chatham et al., 2018).

To investigate how turn‐by‐turn information flow affects response timing (RQ4), we measured the predictability of utterances using a computational approach. For each pair of successive utterances, we calculated how similar or different they were from one another in terms of their semantic content (i.e., cosine similarity). This measure, based on language models (specifically, the all‐mpnet‐base‐v2 model from the Sentence Transformers library (Reimers & Gurevych, 2020)), captures how predictable an utterance is given the previous one. Higher predictability scores indicate that the content of an utterance is more closely related to or follows more naturally from the previous turn. Lower predictability scores indicate novel information being introduced, unexpected responses, or even topic shifts and new conversational directions. By examining how these predictability scores relate to response latencies, we can assess whether children respond differently to more versus less predictable conversational turns, and whether they take longer to produce utterances that represent greater shifts in the conversation.

To investigate how interpersonal adjustment affects turn‐taking (RQ5), we used a multivariate outcome vector autoregression, conditioning the response latencies of both the child and adult on each other's concurrent as well as lagged latencies, including conversational context as a moderator. Varying effects in the two equations were modeled as correlated between children and their interlocutors. This model is thus similar to structural equation modeling approaches, such as the actor–partner interdependence (Cook & Kenny, 2005) and the dyadic coupling (Helm, Sbarra, & Ferrer, 2012) models.

While our analyses focus primarily on children's turn‐taking abilities across contexts, we acknowledge that these behaviors emerge within dyadic interactions where adult interlocutors significantly shape the conversation. In addition to RQ5, which focuses specifically on interpersonal adjustment between children and caregivers, complete parallel analyses of adult behaviors are provided in the Supplementary Materials (Tables S1–S4 and Figs. S1–S4), reflecting our recognition of turn‐taking as fundamentally interpersonal and as an emergent property of the dyadic system. To complement our analysis of interpersonal adjustment, we also conducted Cross‐Recurrence Quantification Analysis (e.g., Dale & Spivey, 2006; Fusaroli, Konvalinka, & Wallot, 2014) to examine nonlinear dynamical coupling patterns and leader‐follower dynamics across interaction contexts. This analysis revealed that social context systematically affects who leads the conversation, with experimenter interactions showing more adult‐led dynamics for the autistic group compared to parent interactions (see Supplementary Materials).

Estimates from the models were reported as mean and 95% Credible Intervals (CI) of the posterior estimates. We calculated evidence ratios (ERs) for our hypotheses in the form of the posterior probability of the directed hypothesis against the posterior probability of all the alternatives; that is, if we expected higher response latencies in autistic children, we would count the posterior samples compatible with this hypothesis and divide them by the number of posterior samples compatible with a null or negative effect. An ER of 5 thus implies that the evidence in favor of the hypothesis is five times greater than the evidence in favor of the alternative model or hypothesis. An ER of “Inf” (short for infinite) implies that all posterior samples are in the direction of the hypothesis, such that the ratio in favor becomes infinite. Throughout the results section, we compare posterior estimates between those in the experimenter and parent conversations, on the one hand, and between those in parent conversations and the matching game (conducted only with parents), on the other hand, in order to ascertain the effect of the familiarity of the interlocutor and the effect of experimental task on response latencies, respectively. Full details on model specification, including weakly informative priors, and model quality checks are available in the Supplementary Materials.

#### 2.4.1 Control analyses

To evaluate the impact of overlaps on response latency patterns for RQ1–RQ3, we conducted two control analyses. First, we re‐ran the models excluding overlaps (i.e., negative latencies) to determine how the inclusion of overlaps in the main analyses potentially influenced our results. Except for specifying a truncated prior indicating no value could be below 0 to facilitate the sampling process, these control models and their specifications were identical to the primary models. Second, to assess the extent of overlap in each group for each task, we ran a multi‐level logistic regression model, with the rate of overlap conditioned on diagnostic group, conversational context, and familiarity of the interlocutor, using the same varying effects structure as in the primary model.

To examine the impact of long pauses on group differences, we conducted a second control analysis. We fitted a simpler version of the primary model where the exponential rate parameter remained constant across contexts, while retaining all other model specifications. This allowed us to evaluate whether failing to account for varying rates of long pauses across contexts affected the estimation of group differences in response timing. Without modeling context‐specific variation in long pauses, the model may overestimate response latencies for children who produced fewer long pauses than expected or underestimate latencies for children who produced more long pauses than expected.

These control analyses provide critical context for interpreting group differences, helping to distinguish the relative roles of overlaps and long pauses in the results and enable better interpretation of the findings in relation to the existing literature. Complete results of these analyses are available in the Supplementary Materials.

In addition to these analyses of overlaps and long pauses, we conducted four further control analyses. First, for our model of information flow, we conducted a control analysis to check whether short, low‐content utterances like backchannels unduly affected the analysis (Corps, Knudsen, & Meyer, 2022) and to make researcher degrees of freedom more explicit in our analyses. We included backchannels in our main analyses as they provide meaningful responsive contributions to conversation that affect the flow and timing of interactions, but acknowledge that different theoretical perspectives exist on their status. While one could argue that backchannels provide cheap signals of common ground and do not require as much cognitive processing to respond to (Dideriksen et al., 2023), we consider them an integral part of the informational landscape, requiring speakers to process contributions and identify appropriate turn transition points. We ran control models with utterances with fewer than three words excluded (see Table S12). Second, due to prior research showing gender differences in turn‐taking dynamics (Cho et al., 2023; Pauline, Weyland, & Kissine, 2024), we conducted an analysis of how response times changed across genders (see Table S7). Third, because the familiarity of the experimenter increased with visits, we checked whether there were any changes in child response latencies according to this increase in familiarity (see Fig. S10). Fourth, as a final overall control analysis, we created surrogate dyads to emulate the baseline turn‐taking that would happen by chance if there were no contingency between adult and child responses (see Table S13). The surrogate pairs were created by pairing the time codes of the caregiver of a different child within the same diagnostic group and visit with the time codes of the child. The aim of this control analysis was to show how our patterns of results could not be reduced to simple differences in vocalization rates and duration but were due to the specific contingency in the interpersonal temporal sequence of vocalizations.

### 2.5 Code availability

All codes and data required to reproduce this research paper are publicly available and documented on OSF and GitHub.

## 3 Results

### 3.1 Social context and diagnostic group (RQ1 and RQ2)

#### 3.1.1 Average response latencies (μ)

**Fig. 2:**
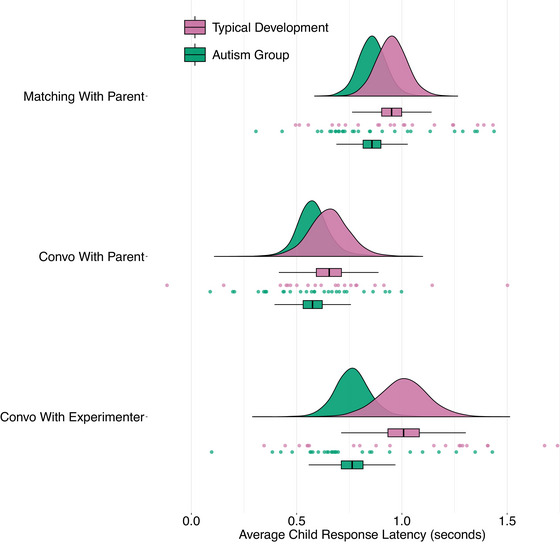
Model estimates for child response latencies across different conversational contexts for autistic children (green) and typically developing children (purple). The points denote participant‐level posterior predictions from the model. The density plots and boxplots show aggregated posterior predictions for the three different social contexts: Matching With Parent, Convo With Parent (Conversations with Parents), and Convo With Experimenter (Conversations with Experimenter). The boxplots show the median (vertical line dividing the box), interquartile range (box edges), and most extreme data points (whiskers) for the aggregated posterior predictions. This figure shows the estimated μ parameter (central tendency) from our ex‐Gaussian model and specifically captures the location of the main distribution after accounting for systematic differences in extended pauses (right tails), which are separately modeled by the beta parameter. Note that these values differ from raw data averages as they account for the hierarchical data structure and separate the distributional components.

The results pertaining to RQ1 and RQ2 are shown in Table 3 and Fig. 2. There were strong differences across social contexts. Children exhibited slower response latencies when conversing with the unfamiliar experimenter than with their parent in both autism (186 ms [2.33, 367.05], ER = 19.3) and typical development groups (353 ms [113, 591], ER = 93.3). The children also produced slower latencies when solving the matching game than when more freely conversing with their parents in both the autism (279 ms [124, 430], ER = 93.3) and typical development groups (297 ms [131, 466], ER = 160.3). Diagnostic group also mattered. Response latencies for autistic children were faster than for those in the typical development group when aggregating across conditions (−136 ms [−267, 0], ER = 18.7). This effect was the strongest in the conversations with the unfamiliar experimenter (−242 ms [−476, −5], ER = 20.1), followed by the matching game (−93 ms [−249, 67], ER = 5, difference in strength from the conversation with the experimenter: −167 ms [−466, 133], ER = 4.6) and the conversations with the parent (−75 ms [−272, 139], ER = 2.8, difference in strength from the matching game: −18 ms [−254, 212], ER = 0.8). Test‐retest reliability of response latency estimates across the sessions was high within each social context, indicating stable individual differences in conversational timing over repeated sessions (see Table 3). Additionally, response latencies exhibited high cross‐context consistency within each child, with those showing faster latencies in one social context tending to be faster in others (Fig. S5). An analysis of how response times changed across genders showed a slight tendency for girls and nonbinary children to be even faster than boys in the autism group: comparisons between the autism and typical development group, however, remained robust (see Table S7). There were also no clear developmental changes in child response latencies according to the increasing familiarity of the experimenter over the course of the multiple visits (see Fig. S10).

**Table 3:** Posterior estimates for child response latencies across individual conditions (Matching With Parent, Convo With Parent [Conversations with Parent], and Convo With Experimenter [Conversations with Experimenter]) and aggregated across conditions (Aggregate) for different model parameters: the Gaussian component (Mean Latency) in milliseconds, proportion of latencies below zero (Overlap Proportion) in proportions, variance (Sigma) in milliseconds, exponential component (Beta, or long pauses) on a log scale, and test‐retest reliability across sessions in correlations.

	Task	Autism Group	Typical Development
**Mean Latency**	Matching With Parent	859 ms [753, 967]	952 ms [833, 1069]
	Convo With Parent	580 ms [460, 716]	655 ms [497, 814]
	Convo With Experimenter	766 ms [630, 908]	1007 ms [810, 1199]
	Aggregate Estimate	735 ms [651, 822]	871 ms [767, 974]
**Overlap**	Matching With Parent	0.22 [0.19, 0.26]	0.22 [0.19, 0.25]
**Proportion**	Convo With Parent	0.23 [0.19, 0.28]	0.17 [0.13, 0.22]
	Convo With Experimenter	0.24 [0.2, 0.29]	0.2 [0.15, 0.24]
	Aggregate Estimate	0.23 [0.21, 0.26]	0.19 [0.17, 0.22]
**Sigma**	Matching With Parent	618 ms [572, 668]	529 ms [482, 578]
	Convo With Parent	541 ms [485, 604]	601 ms [523, 691]
	Convo With Experimenter	954 ms [882, 1030]	898 ms [826, 975]
	Aggregate Estimate	683 ms [649, 720]	658 ms [619, 701]
**Beta**	Matching With Parent	0.19 [0.12, 0.27]	0.28 [0.18, 0.38]
	Convo With Parent	−0.35 [−0.48, −0.23]	−0.31 [−0.45, −0.17]
	Convo With Experimenter	−0.26 [−0.37, −0.14]	−0.18 [−0.33, −0.03]
	Aggregate Estimate	−0.14 [−0.2, −0.08]	−0.07 [−0.15, 0.01]
**Test‐Retest**	Matching With Parent	0.42 [0.27, 0.53]	0.42 [0.24, 0.65]
**Reliability**	Convo With Parent	0.64 [0.44, 0.77]	0.62 [0.35, 0.8]
	Convo With Experimenter	0.36 [0.05, 0.59]	0.66 [0.52, 0.8]

*Note*. All of the parameter estimates listed in this table have an evidence ratio above 40 for a test of difference to null.

#### 3.1.2 Frequency and impact of overlaps

The proportion of overlaps changed across social contexts and diagnostic group (see Table 3). Autistic children were more likely to overlap with adults compared to typically developing children when aggregated across contexts (0.04 [0, 0.08], ER = 21.1). Conversations with parents displayed the biggest group difference (0.06 [0, 0.12], ER = 15.8) followed by conversations with the experimenter (0.05 [−0.01, 0.11], ER = 10.4, strength of difference to conversations with the parent: −0.01 [−0.09, 0.07], ER = 1.5) and the matching game (0.01 [−0.04, 0.05], ER = 1.6, only slightly smaller strength of difference compared to conversations with parents: −0.05 [−0.12, 0.01], ER = 9.2). Removing overlaps did not substantially affect the overall findings on response latencies; the autism group was still faster than the typical development group in the matching game and conversations with the experimenter, albeit not with parents (see Table S5 and Fig. S6).

#### 3.1.3 Variance in response latencies (σ)

Different social contexts were related to a different degree of variance in response latencies (see Table 3). Conversations with an unfamiliar experimenter displayed the highest variance for both the autism group (on log scale: 0.57 [0.43, 0.7], ER = Inf) and the typical development group (0.4 [0.23, 0.57], ER = Inf), followed by the matching game for the autism group (comparison to conversation with parent: 0.13 [0, 0.27], ER = 18.6) and conversation with parent for the typical development group (comparison to matching game: −0.13 [−0.29, 0.03], ER = 9.2). Autistic children displayed a slightly higher degree of variance than typically developing children when aggregated across conditions (on log scale: 0.04 [−0.04, 0.12], ER = 3.5). The strongest between‐group difference was in the matching game (on log scale: 0.16 [0.04, 0.28], ER = 72.5) followed by the conversations with parents (on log scale: −0.1 [−0.28, 0.07], ER = 5.1, difference to conversations with parents on log scale: 0.26 [0.05, 0.48], ER = 40.3) and conversations with the experimenter (on log scale: 0.06 [−0.06, 0.17], ER = 4.3, difference to parent conversations: on log scale: 0.16 [−0.05, 0.38], ER = 9).

#### 3.1.4 Long pauses (β)

The prevalence of long pauses (i.e., the exponential tail) was influenced by social context and diagnostic group (see Table 3). The matching game displayed a higher tendency for long pauses than the conversations with the unfamiliar experimenter in both the autism group (0.55 [0.42, 0.69], ER = Inf) and typical development group (0.59 [0.42, 0.76], ER = Inf). Children in both the autism group and typical development group had a slightly lower tendency to include long pauses when conversing with parents than when conversing with the experimenter (Autism Group: 0.1 [−0.07, 0.27], ER = 5.5; Typical Development Group: 0.13 [−0.06, 0.33], ER = 6.7). Autistic children produced fewer long pauses when aggregated across conditions (on a log scale: −0.07 [−0.17, 0.03], ER = 6.5) and during both the matching game (−0.08 [−0.21, 0.04], ER = 6.8) and experimenter conversations (−0.08 [−0.26, 0.11], ER = 3.2), but not during the conversations with parents (−0.04 [−0.23, 0.15], ER = 1.9). There were no reliable differences in the strength of group differences across social contexts.

### 3.2 Individual differences (RQ3)

**Fig. 3:**
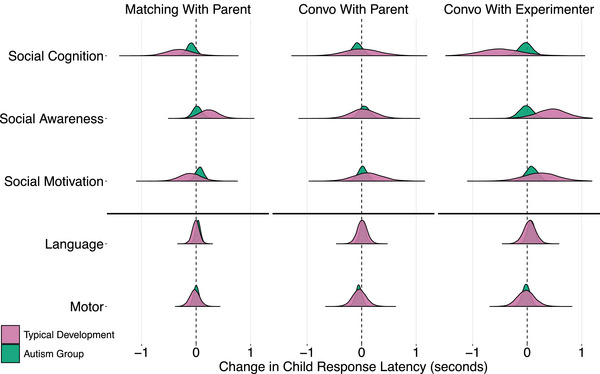
Model estimates for change in child response latencies as a function of one standard deviation increase in each measure across the three social contexts: Matching Game, Convo With Parent (Conversations with Parents), and Convo With Experimenter (Conversations with Experimenter). The boxplots show the median (vertical line dividing the box), interquartile range (box edges), and most extreme datapoints (whiskers) for the aggregated posterior predictions. The top three measures (i.e., Social Cognition, Social Awareness, and Social Motivation) come from the SRS‐2, whereas Language Skills refer to CELF‐5 and Motor Skills refer to VAB‐3.

The results pertaining to RQ3 are shown in Table 4 and Fig. 3. Estimates denote the change in child response latencies as a function of one standard deviation increase in each measure. Our control analyses excluding overlaps displayed analogous findings across all measures of individual differences, as shown in Table S10 and Fig. S8.

**Table 4:** Posterior estimates for how child response latencies change across individual conditions (Matching With Parent, Conversations With Parent, and Conversations With Experimenter) and all conditions (Aggregate Estimate) as a function of each of the types of skills listed below (Motor, Cognitive Skills, Language Skills, Social Awareness, and Social Motivation).

Skill	Task	Autism Group	Typical Development
**Social Cognition**	Matching With Parent	−89 ms [−233, 52]	−301 ms [−690, 114]
	Convo With Parent	−78 ms [−238, 84]	8 ms [−511, 554]
	Convo With Experimenter	−27 ms [−234, 179]	−478 ms [−1098, 169]
	Aggregate Estimate	−65 ms [−185, 57]	−257 ms [−592, 79]
**Social Awareness**	Matching With Parent	19 ms [−127, 168]	228 ms [−62, 517]
	Convo With Parent	52 ms [−108, 216]	29 ms [−354, 412]
	Convo With Experimenter	−19 ms [−232, 198]	452 ms [−10, 910]
	Aggregate Estimate	18 ms [−107, 146]	236 ms [−14, 486]
**Social Motivation**	Matching With Parent	65 ms [−63, 193]	−105 ms [−424, 218]
	Convo With Parent	17 ms [−124, 157]	117 ms [−320, 567]
	Convo With Experimenter	82 ms [−102, 268]	248 ms [−269, 773]
	Aggregate Estimate	55 ms [−53, 164]	87 ms [−187, 370]
**Language**	Matching With Parent	35 ms [−42, 113]	−6 ms [−113, 102]
	Convo With Parent	21 ms [−68, 112]	10 ms [−140, 163]
	Convo With Experimenter	72 ms [−41, 185]	45 ms [−140, 231]
	Aggregate Estimate	43 ms [−23, 110]	17 ms [−80, 116]
**Motor**	Matching With Parent	4 ms [−79, 88]	−26 ms [−180, 128]
	Convo With Parent	−54 ms [−152, 45]	−40 ms [−251, 175]
	Convo With Experimenter	−19 ms [−141, 101]	−19 ms [−278, 252]
	Aggregate Estimate	−23 ms [−96, 51]	−29 ms [−169, 113]

*Note*. The top three measures (i.e., Social Cognition, Social Awareness, and Social Motivation) came from the SRS‐2 scale, Language skills from CELF‐5 scale, and Motor skills from VAB‐3. The estimates denote the change in child response latency as a function of one standard deviation increase in the skills of the children.

#### 3.2.1 Social cognition

Children with higher social cognition skills displayed faster responses across groups and contexts, although the effect was much more evident in typically developing children (Autism Group: −65 ms [−185, 57], ER = 4.3; Typical Development: −257 ms [−592, 79], ER = 8.4; Difference: 192 ms [−171, 551], ER = 4.3). The influence of social cognition changed according to social context. The response latencies of typically developing children—but not of autistic children—when compared to the conversations with parents showed a bigger relation to social cognition in the conversation with the unfamiliar experimenter (Difference: −486 ms [−1253, 300], ER = 5.8) as well as in the matching game (−309 ms [−902, 267], ER = 4.4).

#### 3.2.2 Social awareness

Typically developing children—but not autistic children—displayed a strong association between social awareness and responses across contexts (Autism Group: 18 ms [−107, 146], ER = 1.4; Typical Development: 236 ms [−14, 486], ER = 16.1; Difference: −219 ms [−498, 59], ER = 9.4). The effect of social awareness for the typical development group was most evident in the conversation with the experimenter compared to in the matching game (423 ms [−131, 976], ER = 8.6), as well as in the matching game compared to the conversation with parents (199 ms [−224, 624], ER = 3.7).

#### 3.2.3 Social motivation

The relation between social motivation and response latencies was small and unsystematic across groups and contexts. Autistic children with higher social motivation showed a consistent but only very slight slowdown (55 ms [−53, 164], ER = 4), especially in interactions with the experimenter (82 ms [−102, 268], ER = 3.4). Typically developing children only displayed the same effect in the conversation with an unfamiliar experimenter (248 ms [−269, 773], ER = 3.7) and the opposite effect in the matching game with parents (−105 ms [−424, 218], ER = 2.4).

#### 3.2.4 Language skills

Similarly, there was only anecdotal evidence for a relation between language skills and response latencies across groups and contexts. We restrict the reporting here to the strongest absolute effect and direct the reader to Table 4 to see the other effects. Autistic children with higher language skills showed an overall small tendency for slower response times compared to the typical development group, but there was no clear evidence for a difference between groups (Autism Group: 43 ms [−23, 110], ER = 5.8; Typical Development: 17 ms [−80, 116], ER = 1.6, Difference: 26 ms [−92, 144], ER = 1.8).

#### 3.2.5 Motor skills

Lastly, there was likewise only anecdotal evidence for a relation between motor skills and response latencies across groups and contexts, and we direct the reader to Table 4 to see other effects not reported here. Children with higher motor skills had slightly faster response latencies in the autism group (−23 ms [−96, 51], ER = 2.4), as well as in the typical development group (−29 ms [−169, 113], ER = 1.8), but there was no evidence for a difference between groups (5 ms [−156, 165], ER = 1.1).

### 3.3 Predictability (RQ4)

**Fig. 4:**
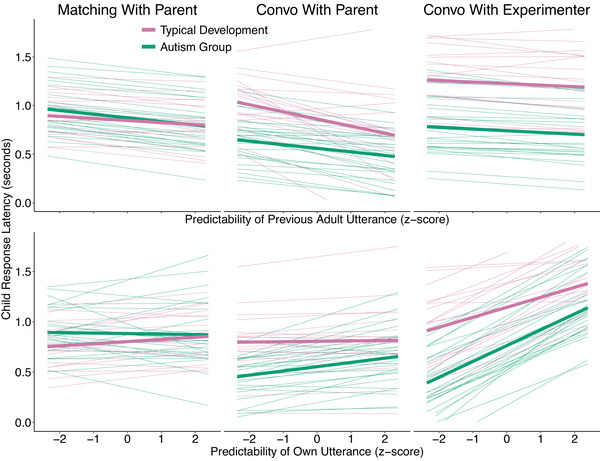
Model estimates for how child response latencies change as a function of the predictability of the previous adult response latency (top) and the predictability of their own utterance (bottom) across the three social contexts: Matching Game, Convo With Parent (Conversations with Parents), and Convo With Experimenter (Conversations with Experimenter). The cosine similarity score here represents scaled values (i.e., one unit increase equals one standard deviation increase in cosine similarity). The faded lines are posterior predictions from the model for individual child participants in the study, whereas the thicker lines are average predictions.

The results pertaining to RQ4 are shown in Table 5 and Fig. 4. Our control analyses excluding overlaps and short utterances displayed equivalent findings, as shown in Table S12.

**Table 5:** Posterior estimates for how child response latencies change across individual conditions (Matching With Parent, Conversations with Parent (Convo with Parent), and Conversations with Experimenter (Convo With Experimenter) and all conditions (Aggregate Estimate) as a function of the predictability of the previous adult utterance and child utterance.

	Tasks	Autism Group	Typical Development
**Predictability**	Matching With Parent	−39 ms [−65, −11]	−17 ms [−53, 21]
**of Previous**	Convo With Parent	−37 ms [−71, −1]	−70 ms [−127, −11]
**Adult Utterance**	Convo With Experimenter	−10 ms [−48, 30]	−9 ms [−63, 45]
	Aggregate Estimate	−28 ms [−48, −9]	−32 ms [−61, −3]
**Predictability**	Matching With Parent	−4 ms [−39, 32]	25 ms [−14, 68]
**of Own**	Convo With Parent	44 ms [7, 80]	6 ms [−30, 42]
**Utterance**	Convo With Experimenter	167 ms [122, 211]	119 ms [56, 182]
	Aggregate Estimate	69 ms [47, 91]	50 ms [22, 79]

*Note*. The estimates denote the change in child response latency as a function of one standard deviation increase in the predictability of the utterance.

#### 3.3.1 Predictability of previous adult utterance

There was strong evidence that both autistic and typically developing children responded faster to more predictable utterances from their interlocutor (Autism Group: −28 ms [−48, −9], ER = 101; Typical Development: −32 ms [−61, −3], ER = 25.2; Difference: 4 ms [−32, 40], ER = 1.3). Conversations with parents generated bigger effects of predictability across groups compared to those with the experimenter (Autism Group: −27 ms [−80, 25], ER = 4; Typical Development: −61 ms [−138, 19], ER = 9.1). There were weaker effects in the matching game compared to in conversations with the parent, but only for the typically developing children (Autism Group: −2 ms [−48, 42], ER = 0.9; Typical Development: 53 ms [−14, 121], ER = 9.3).

#### 3.3.2 Predictability of own utterance

There was strong evidence that both autistic and typically developing children responded faster if they were planning a low predictability utterance than a high predictability utterance (Autism Group: 69 ms [47, 91], ER = Inf; Typical Development: 50 ms [22, 79], ER = 383.6, Difference: 18 ms [−18, 54], ER = 4.1). The effects were more evident in conversations with the unfamiliar experimenter compared to with parents (Autism Group: −123 ms [−181, −66], ER = 4999; Typical Development: −113 ms [−186, −41], ER = 121), and lower in the matching game compared to conversations with parents for the autism group but not for the typical development group (Autism Group: −48 ms [−99, 3], ER = 15.6; Typical Development: 19 ms [−35, 76], ER = 2.4) .

### 3.4 Interpersonal adjustment (RQ5)

**Fig. 5:**
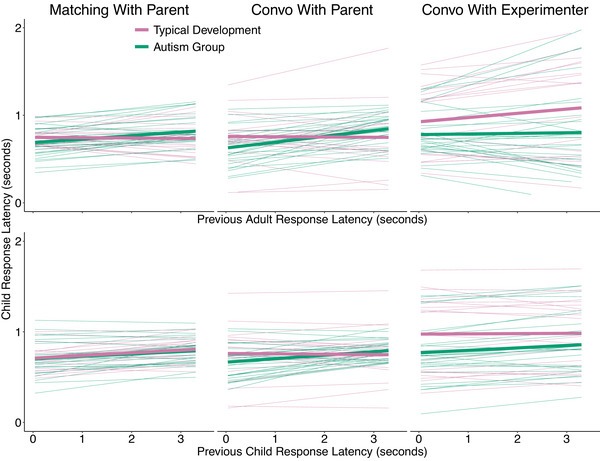
Model estimates for how child response latencies change with the latency of the previous adult response latency (top) and the previous child response latency (bottom) across the three social contexts: Matching Game, Convo With Parent (Conversations with Parents), and Convo With Experimenter (Conversations with Experimenter). The faded lines are posterior predictions from the model for individual child participants in the study, whereas the thicker lines are average predictions.

The results pertaining to RQ5 are shown in Table 6 and Fig. 5. The estimates denote the change in child response latency as a function of a 1‐s increase in the response latency of the previous utterance. The analysis revealed a disparity between autistic and typically developing children in both interpersonal and self‐adjustment of response latencies across social contexts. When looking at the adult interlocutors, we see weak positive effects across different social contexts and no robust differences between the autism and typical development group (see Fig. S4 and Table S4).

**Table 6:** Posterior estimates for how child response latencies change across individual conditions (Matching With Parent, Conversations With Parent, and Conversations With Experimenter) and all conditions (Aggregate Estimate) as a function of the latency of the previous adult utterance (Interpersonal Adjustment) and previous child response latency (Self‐Adjustment)

	Tasks	Autism Group	Typical Development
**Interpersonal**	Matching With Parent	28 ms [9, 47]	13 ms [−14, 39]
**Adjustment**	Convo With Parent	68 ms [32, 107]	−6 ms [−55, 40]
	Convo With Experimenter	18 ms [−34, 71]	22 ms [−31, 74]
	Aggregate Estimate	38 ms [16, 60]	10 ms [−17, 35]
**Self‐**	Matching With Parent	24 ms [7, 41]	31 ms [7, 54]
**Adjustment**	Convo With Parent	46 ms [7, 89]	12 ms [−36, 64]
	Convo With Experimenter	35 ms [0.82, 69.39]	−5 ms [−46, 38]
	Aggregate Estimate	35 ms [17, 54]	13 ms [−10, 36]

*Note*. The estimates denote the change in child response latency as a function of a 1‐s increase in the response latency of the previous utterance.

#### 3.4.1 Interpersonal adjustment

Autistic children demonstrated a small but reliable positive adjustment to their interlocutors' response latencies, whereas typically developing children did not (Autism Group: 38 ms [16, 60], ER = 374; Typical Development: 10 ms [−17, 35], ER = 2.8; Difference: 29 ms [−6, 63], ER = 11.1). Autistic children displayed a higher degree of adjustment in conversations with parents compared to in the matching game (Difference: −40 ms [−85, 1], ER = 17.4), as well as compared to in conversations with the experimenter (Difference: −50 ms [−118, 17], ER = 8.6). In comparison, typically developing children displayed no effect of the latency of the previous adult turn, and no clear differences across contexts.

#### 3.4.2 Self‐adjustment

Children in both groups displayed a small positive adjustment to their own previous response latency, but the autistic children were more driven by their own previous response latency when aggregated across social contexts (Autism Group: 35 ms [17, 54], ER = 749; Typical Development: 13 ms [−10, 36], ER = 4.3; Difference: 23 ms [−8, 52], ER = 8.7). Children in the autism group adapted slightly less to their own response latencies in the matching game compared to when conversing with their parents (Difference: −22 ms [−66, 19], ER = 3.8), but there were no differences of this sort in the typical development group (Difference: 19 ms [−38, 72], ER = 2.7). The strongest difference between the groups was in conversations with the experimenter (Difference: 40 ms [−14, 94], ER = 7.7), followed by conversations with parents (Difference: 34 ms [−29, 99], ER = 4.4), and there were no differences in the matching game (Difference: −6 ms [−35, 23], ER = 1.8).

## 4 Discussion

In this study, we set out to challenge the idea of response latencies in turn‐taking as a monolithic individual trait by investigating turn‐taking as the interplay of individual differences, information flow, and interpersonal dynamics—all of which are shaped by social context. We argue that a fuller understanding of child–adult turn‐taking dynamics requires consideration of how results vary across multiple sessions and in different social contexts (RQ1), within atypical and typical development (RQ2), as well as how these dynamics interact with individual differences (RQ3) and turn‐by‐turn variations due to informational content (RQ4) and shared tempo (RQ5).

Our findings indicated that inter‐turn response latencies were consistent across repeated measures *within social contexts*, but exhibited substantial differences across social contexts (RQ1). Conversations with a familiar interlocutor enabled faster turn‐taking (i.e., faster child response latencies with a parent compared to an experimenter), and more structured tasks slowed down the conversation (i.e., slower responses in the matching game compared to conversations with their parent). We also found substantial differences between diagnostic groups (RQ2) modulated by social context; autistic children produced faster (more overlaps and shorter gaps) and more variable inter‐turn response latencies compared to typically developing children —and these group differences were particularly salient in interactions with the unfamiliar interlocutor. Turn‐taking dynamics were also modulated by individual differences among children (RQ3). Higher social cognition skills were related to faster response latencies in both groups, whereas children with higher social awareness and motivation exhibited slight slowdowns in responses in the typical development and autism group, respectively. Social context also shaped how turn‐by‐turn information flow related to response latencies (RQ4). The more predictable the interlocutor's previous utterance and the less predictable the child's own coming utterance, the faster the response. Yet, with unfamiliar experimenters, the predictability of the other's utterance ceased to play a role, and the predictability of the child's own utterance became a stronger predictor. Analogously, social context modulated the turn‐taking dynamics of intra‐ and interpersonal adjustment (RQ5). Only the autism group adjusted their response times to those of the adult's preceding turn—and only in interactions with their parents. Both groups displayed a weak but positive autocorrelation of their own successive response latencies, but less so when interacting with the experimenter.

Our findings demonstrate how deconstructing turn‐taking into its constituent dimensions can reveal new insights on conversational dynamics. By explicitly including individual differences, moment‐by‐moment information flow, and interpersonal rhythms—and their relation to changes in social context—we identified how the interplay among these dimensions can shape inter‐turn response latencies. This multidimensional approach proved particularly valuable in theorizing about two key phenomena: the faster response latencies of autistic children and the systematic effects of interlocutor familiarity. We view these timing patterns not as products of simple linear causation but as emergent properties that differentially scaffold interaction coherence across contexts. In the following sections, we consider individual and interpersonal mechanisms that may underlie the faster responses of autistic children across social contexts. We then examine how interlocutor familiarity differentially shapes the interplay of group differences, individual characteristics, and turn‐by‐turn processes, providing a more nuanced understanding of its impact on turn‐taking dynamics. Together, our approach reveals that turn‐taking is a multicomponential construct that is shaped by individual differences, cognitive processing demands, and interpersonal adaptations—all of which are systematically modulated by the contextual demands of different conversational settings.

### 4.1 Why do we see faster responses in autism?

Whereas earlier studies have found that autistic individuals exhibit slower response latencies (about 300 ms) than neurotypicals (Nguyen et al., 2022; Ochi et al., 2019), we found that autistic children are faster by around 140 ms (replicating Fusaroli et al., 2025, and to a certain extent Wehrle et al., 2023). Understanding the source of this effect requires unpacking multiple factors. In conversations with parents, autistic children tended to produce more overlaps than neurotypical children, which, in turn, may explain why they respond faster (see also Fusaroli et al., 2025). However, even after excluding overlaps, autistic children still had shorter gaps in the matching game and in conversations with the experimenter (see Table S5). Autistic children also tended to produce fewer long pauses than typically developing children in the matching game; not modeling the long tail removes the group difference in the matching game, but not in the other conversations (see Table S9). Taken together, the findings observed in the conversation with parents and in the matching game could be discrepant from previous literature at least partially because of the differences in the statistical treatment of the data. Our findings for interactions with the unfamiliar interlocutor, on the other hand, continue to yield a clear group difference even when removing overlaps and long pauses. We argue that the explanation for this remaining difference is to be found in the underlying mechanisms of turn‐taking. Crucially, conversations are both the expression of the individual interlocutors involved and of the interpersonal dynamics emerging between them. While autistic children might exhibit atypical processing and behaviors, adults might also bring different expectations to a conversation with autistic children and accordingly provide different scaffolding and reactions to them than to typically developing children (Ferjan Ramírez, Lytle, & Kuhl, 2020). Consequently, we discuss possible complementary explanations of group differences in terms of both child and adult behaviors in the interaction (Fusaroli, Weed, Rocca, Fein, & Naigles, 2023a, 2023b).

#### 4.1.1 Individual mechanisms: Child executive function and impulsivity

Autistic children are often attributed a large suite of differences in socio‐cognitive and other abilities (Frith & Happé, 1994; Fletcher‐Watson & Happé, 2019). In our analyses, we included individual differences in social cognition, motivation and awareness, language and motor skills, which we had hypothesized to be directly involved in the development of turn‐taking skills (RQ2). Nonetheless, group differences remained, even when stratifying by those individual differences. A complementary mechanism generating the prevalence of overlaps, short gaps, and scarcity of long pauses could be located in the atypical executive function sometimes observed in autism (Hlavatá, Kašpárek, Linhartová, Ošlejšková, & Bareš, 2018; Russell, 1997), and the perhaps related high comorbidity with Attention Deficit Hyperactivity Disorder and its impulsivity (Bougeard, Picarel‐Blanchot, Schmid, Campbell, & Buitelaar, 2021; Carrascosa‐Romero & De Cabo‐De La Vega, 2015; Fusaroli et al., 2023). Atypical executive function is often expressed as impulsivity and lack of inhibition (Geurts, van den Bergh, & Ruzzano, 2014; Hughes, Russell, & Robbins, 1994; Hill, 2004; Hlavatá et al., 2018). For instance, as an utterance unfolds, each new word becomes more and more predictable. A child with high impulsivity might have a lower threshold for predictability, be more likely to lose attention, or just be generally impatient and jump in earlier. This might involve increased backchannels, or interrupting and neglecting the social nicety of letting the other finish (Cola et al., 2022; Dobbinson, Perkins, & Boucher, 1998). Such a tendency to overlap might be further emphasized in the relaxed atmosphere of a conversation with a caregiver, where indeed the propensity to overlap is particularly high for autistic children.

#### 4.1.2 Interpersonal mechanisms: Adult expectations and predictability

Group differences in children's response latencies may also be driven by the behaviors of the adult in the interaction, and the feedback cycles of adjusting to each other (Cox et al., 2023; Dingemanse et al., 2023; Fusaroli et al., 2014; Fusaroli et al., 2019). For example, while adult interlocutors did display comparable response latencies, overlap proportions, long pauses, and degrees of variability across the two groups (cf., Tables S1 and S6), they also produced speech with a higher average turn‐level predictability when interacting with autistic children (see Table S11). This is in line with previous work showing that parents of autistic children produce shorter utterances and employ a smaller vocabulary (Fusaroli et al., 2019). Thus, autistic children might not be more impulsive and more likely to interrupt their interlocutor; they may simply be reacting to predictable information in a typical way—and if typically developing children were to engage with the same predictable language, they may behave in similar ways to the autistic children.^1^ Differences in adult behaviors might be due to different expectations about their children on the basis of diagnostic status and individual differences—but also due to other attested differences in children's behaviors—for example, atypical prosodic, verbal, or multimodal patterns (Fusaroli et al., 2019; Fusaroli, Lambrechts, Bang, Bowler, & Gaigg, 2017; Fusaroli et al., 2022; Rybner et al., 2022). Further, adults might be sensitive to interventions emphasizing how they need to produce good examples of social interactions and well‐composed language (Ferjan Ramírez et al., 2020; Huber, Ferjan Ramírez, Corrigan, & Kuhl, 2023). Thus, a typically developing child overlapping with the adult might be quickly given the floor; while an autistic child might not, and the adult might keep speaking until the utterance reaches a possible conclusion.

### 4.2 Turn‐taking as shaped by interlocutor familiarity

The familiarity of the interlocutor (i.e., caregiver vs. experimenter) played a crucial role in shaping not only the children's response latencies, but also how differences by diagnostic group, individual characteristics, and turn‐by‐turn dynamics played out; more so than the conversational task did. Engaging in verbal exchanges with unfamiliar interlocutors fundamentally differs from doing so with familiar interlocutors. For example, college students responded faster when interacting with unfamiliar interlocutors to communicate active listening and engagement (Templeton et al., 2022, 2023). The children in this study, on the contrary, slowed their response latencies (by around 200 ms in autism and 350 ms in typical development), varied them more, and produced long pauses more frequently when interacting with the unfamiliar experimenter than with the familiar parent.

One possible explanation is that interactions with unfamiliar interlocutors are much more challenging for children. While adults can be prompted by the increased social pressure to give at least the impression of a prompt and smooth turn‐taking, this might be too tall an order for children. Unfamiliarity brings the challenge of additional unpredictability and uncertainty to the interaction. The interlocutors do not share a history of interactional routines, and that makes inference and anticipation more challenging (De Marchena & Eigsti, 2016; Kimhi, Shoam‐Kugelmas, Agam Ben‐Artzi, Ben‐Moshe, & Bauminger‐Zviely, 2014; Pickering & Garrod, 2021; Tylén et al., 2013; Ying Sng, Carter, & Stephenson, 2018). We observe, for example, that the average predictability of the speech content in adult utterances was lower with unfamiliar than in familiar interlocutors (see Table S11), and this measure of predictability does not even account for the accumulated common ground and the multimodal and social cues that children might have learned to pick up on in familiar adults throughout years of interactions.

The challenges involved in increased uncertainty might also be reflected in the relative lack of responsiveness to the utterance content of the unfamiliar interlocutor. The predictability of the other's utterances and previous response latencies mattered less when interacting with unfamiliar interlocutors—instead, response latencies were relatively more affected by the predictability of their own production. With increased uncertainty, children seem less able to promptly engage and pick up on cues. The finding that children responded faster when planning less predictable utterances may similarly suggest an information‐theoretic mechanism: when preparing to contribute novel or unexpected information (low predictability), there may be greater communicative urgency to quickly take the floor. This urgency appears heightened in unfamiliar contexts, where establishing informational common ground becomes particularly important. Conversely, when planning more predictable responses that merely continue or affirm the previous speaker's contribution, the reduced information value may decrease the perceived pressure to respond rapidly.

Beyond these content‐based effects, our recurrence analysis provides further support for these interpersonal mechanisms by revealing systematic differences in who is setting the conversational tempo across contexts. Our Cross‐Recurrence Quantification Analysis (see Fig. S21) showed that interactions with unfamiliar experimenters were characterized by differential dynamics depending on diagnostic group: autistic children showed increasing trends toward adult‐led dynamics, while typically developing children exhibited less adult‐led structure. These differential responses to unfamiliar interlocutors may partially explain why group differences in turn‐taking were most pronounced in this context. When the two groups engage with fundamentally different conversational structures in response to unfamiliar adults, this may amplify the impact of individual differences in sociocognitive skills, as children must navigate not only more unpredictable content but also varying degrees of conversational control.

In conversations with unfamiliar experimenters, we see the largest difference between autistic and typically developing children across social contexts, with typically developing but not autistic children showing a big relative slowdown. This is in line with complementary evidence that autistic children do not display as large a reaction as typically developing children in the presence of unfamiliar individuals compared to familiar ones (Stefanatos & Baron, 2011). For example, in contrast to typically developing children, autistic children show no difference in gaze aversion when interacting with familiar and unfamiliar interlocutors (Doherty‐Sneddon et al., 2013), no difference in brain activation in response to familiar versus unfamiliar face stimuli (Dawson et al., 2002), and less flexibility in their behavioral repertoire across different social tasks (Forgeot d'Arc et al., 2020; Nadig, Seth, & Sasson, 2015).

Further, sociocognitive skills seem to have a stronger impact on response latencies exactly when facing unfamiliar interlocutors. In typical development, the higher the sociocognitive skills and the lower the social awareness, the faster the response latencies—both due to increased overlaps (cf., Fig. S8) and shorter gaps (cf., Table S10). Social awareness might make the children more cautious and careful in the presence of a stranger, and perhaps make them worried of cutting them off or just slower, while sociocognitive skills might make them better at anticipating when an utterance is tapering to a conclusion. However, we did not see the same patterns in autistic children. Higher social motivation did slow autistic children down through fewer overlaps and longer gaps (cf., Fig. S8)—perhaps again in cautious consideration of the unfamiliar situation, and them trying to do their best at steering clear from the interlocutor's utterances—but higher sociocognitive skills did not speed them up.

The marked diagnostic difference in the impact of sociocognitive skills could be caused by many diverse mechanisms. Autistic children tend to have lower sociocognitive skills (see Table 1) and thus might not be able to fully take advantage of social cues; or the measure of sociocognitive skills might be more sensitive to differences in the lower values (more represented in the neurotypical than in the autistic group). Alternatively, anxiety and/or impulse control issues—often associated with autism (Bougeard et al., 2021; Carrascosa‐Romero & De Cabo‐De La Vega, 2015)—might make them less sensitive to social cues they might otherwise catch to various degrees in more familiar settings. Additionally, different conversational contexts impose varying psycholinguistic processing demands.

In sum, familiarity seems to have a strong impact on turn‐taking and interact with individual and group differences as well as turn‐by‐turn dynamics. As unfamiliarity emphasizes differences by diagnosis and sociocognitive skills, it can be considered a highly informative context to assess children's turn‐taking skills. However, the findings also emphasize that atypical performance with unfamiliar conversation partners only tells part of the story about children's ability to engage with their everyday social context.

### 4.3 Limitations and outlook

Since child populations, and particularly autistic children, are highly heterogeneous across different ages, much work still needs to be done to assess the generalizability of our work. For instance, younger (2–5 years) children show similar but not identical patterns (e.g., with linguistic skills being better predictors of response latencies than in the current study (Fusaroli et al., 2025)). Despite matching groups on age at the group level, developmental differences across the relatively wide age range (6–17 years for the autism group and 6–15 years for typical development) of our participants might influence turn‐taking dynamics in ways not fully captured by our analyses. Future work with larger samples could investigate how developmental trajectories of turn‐taking differ across diagnostic groups and how they interact with social context demands.

As we start measuring more fine‐grained individual differences, we focused on skills traditionally related to language acquisition and turn‐taking: sociocognitive, linguistic, and motor skills. Yet, the current findings exhibited substantial heterogeneity in response latencies beyond differences in such skills (e.g., the remaining population‐level differences between the autism and typical development groups). Network approaches to neurodevelopmental conditions highlight that individuals sharing the same diagnosis might present nonoverlapping clinical profiles, and a better approach would be to rather fully decompose the diagnostic category into a wide range of interacting cognitive and clinical characteristics (Borsboom & Cramer, 2013; Fried & Cramer, 2017; Rocca, Pistilli, Maheshwari, & Fusaroli, 2024), including measures of impulsivity. Further, measures of anxiety level could also be valuable, given the findings that unfamiliar interlocutors elicited markedly different response latency patterns (Vasa, Keefer, McDonald, Hunsche, & Kerns, 2020). More work is needed in articulating how a wider range of individual differences—from sensory overload to information integration processes and attention (Murray, Lesser, & Lawson, 2005; Pellicano & Burr, 2012; Simonsen et al., 2021)—might be involved in the mechanisms of turn‐taking.

We also need to move toward a more interpersonal perspective on social interactions: how are adults shaping their behaviors and reactions when interacting with autistic children—and how are autistic children in turn reacting to this? A crucial component to achieving these steps will also be to integrate a detail‐oriented qualitative analysis of what goes on in the utterances and at turn transitions (Bottema‐Beutel et al., 2022; Sacks, Schegloff, & Jefferson, 1974), including the social context, contingency, and the diverse functions of gaps and overlaps. Besides generating novel research questions, qualitative analysis can be used to inform measurement and statistical modeling. One example is the measurement of utterance predictability. Whereas our measure was based purely on a short context of verbal data, utterance predictability is likely to be driven by several multimodal cues (e.g., gaze, prosody), as well as longer‐term construction of common ground (Bögels & Torreira, 2021; Local & Walker, 2012; Schaffer, 1983). Our current measure of response latency is likewise agnostic as to whether the previous utterance has concluded or been interrupted or as to whether the current utterance is an answer, a completion, or a nonsequitur. Careful qualitative work has much to contribute to the understanding of the complexities and mechanisms of turn‐taking (Bottema‐Beutel et al., 2022).

## 5 Conclusion

Behind turn‐taking lies a complex mutual interaction, intertwining contextual, individual, and turn‐by‐turn processes (Harrist & Waugh, 2002; Schertz, Odom, Baggett, & Sideris, 2018). Here, we outline the central components underlying turn‐taking: individual differences, properties of the utterance, and interpersonal dynamics, whose impact is deeply shaped by social context: the purpose of the conversation and the relation to the interlocutor. This framework construes turn‐taking as immersed in a social context with different affordances for interactions. By considering these components, we not only need to reevaluate the group differences in turn‐taking between neurotypical children and children with autism, but also the construct of turn‐taking latency as a monolithic individual trait, or even indicator of social competence altogether. The picture we sketch of turn‐taking is complex, but likely incomplete and more work is needed to develop this framework, including multimodality and multiple timescales of coordination.

## Code and data availability statement

All codes, data, and materials required to reproduce this research paper are publicly available and documented on OSF and GitHub.

## Supporting information

Data S1

## Note

1 Note that there is no reliable difference in reactions to predictability between groups (cf. Section 3.3.1), but that analysis focuses on turn‐level predictability, and here we are talking about within‐turn, word‐level predictability.
