# Supplementary material for: Social Context Matters for Turn‐Taking Dynamics: A Comparative Study of Autistic and Typically Developing Children
Source: Cogn Sci. 2025 Oct 13;49(10):e70124. doi: 10.1111/cogs.70124 (PMC12517399; doi:10.1111/cogs.70124)
Supplement: Supplementary file 1 — Data S1 [file COGS-49-e70124-s001.pdf]

Wehrle, S., Cangemi, F., Janz, A., Vogeley, K., & Grice, M. (2023). Turn-timing in conversations between autistic adults: Typical short-gap transitions are preferred, but not achieved instantly. *Plos one*, *18*(4), e0284029.

West, K. L., & Iverson, J. M. (2021). Communication changes when infants begin to walk. *Developmental science*, *24*(5), e13102.

Wiig, E. H., Secord, W. A., & Semel, E. (2013). *Clinical evaluation of language fundamentals: CELF-5.* Pearson.

Wilson, M., & Wilson, T. P. (2005). An oscillator model of the timing of turn-taking. *Psychonomic bulletin & review*, *12*, 957–968.

Ying Sng, C., Carter, M., & Stephenson, J. (2018). A systematic review of the comparative pragmatic differences in conversational skills of individuals with autism. *Autism & Developmental Language Impairments*, *3*, 2396941518803806.

Zamm, A., Pfordresher, P. Q., & Palmer, C. (2015). Temporal coordination in joint music performance: effects of endogenous rhythms and auditory feedback. *Experimental Brain Research*, *233*, 607–615.

S.1. Supplementary Materials

Our Supplementary Materials include additional results on adult response latencies, test-retest reliability, control analyses as well as model information and quality checks:

- S.1.1 Adult Results 2
  - S.1.1.1 Average Response Latencies 2
  - S.1.1.2 Individual Socio-Cognitive Differencs, Adults 4
  - S.1.1.3 Predictability, Adults 6
  - S.1.1.4 Shared Tempo, Adults 8
- S.1.2 Test-Retest Reliability 10
- S.1.3 Control Analyses 12
  - S.1.3.1 Child Latency With No Overlaps 12
  - S.1.3.2 Adult Latency With No Overlaps 14
  - S.1.3.3 Child Latency Across Gender 16
  - S.1.3.4 Child Latency With No Sigma and No Beta 17
  - S.1.3.5 Child Latency With Sigma But No Beta 18
  - S.1.3.6 Child Overlaps according to Individual Differences 19
  - S.1.3.7 Child Individual Differences With No Overlaps 21
  - S.1.3.8 Predictability 22
  - S.1.3.9 Child Latency With Predictability, No Short Utterances 23
  - S.1.3.10 Child Latency With Increasing Familiarity 25
  - S.1.3.11 Child Latency For Surrogate Pairs 26
- S.1.4 Model Information and Quality Checks 28
  - S.1.4.1 Choice of Priors 28
  - S.1.4.2 Prior and Posterior Predictive Checks 29
  - S.1.4.3 Prior-Posterior Update Plots 32
- S.1.5 Time Code Reliability 36
- S.1.6 Recurrence Analysis 38
- S.1.7 Research Assistants 42
  - S.1.7.1 Assignment of Research Assistants 42
  - S.1.7.2 Effects of Research Assistant 43
- S.1.8 Recording Quality Assessment 45

S.1.1. Adult Results

S.1.1.1 Average Response Latencies

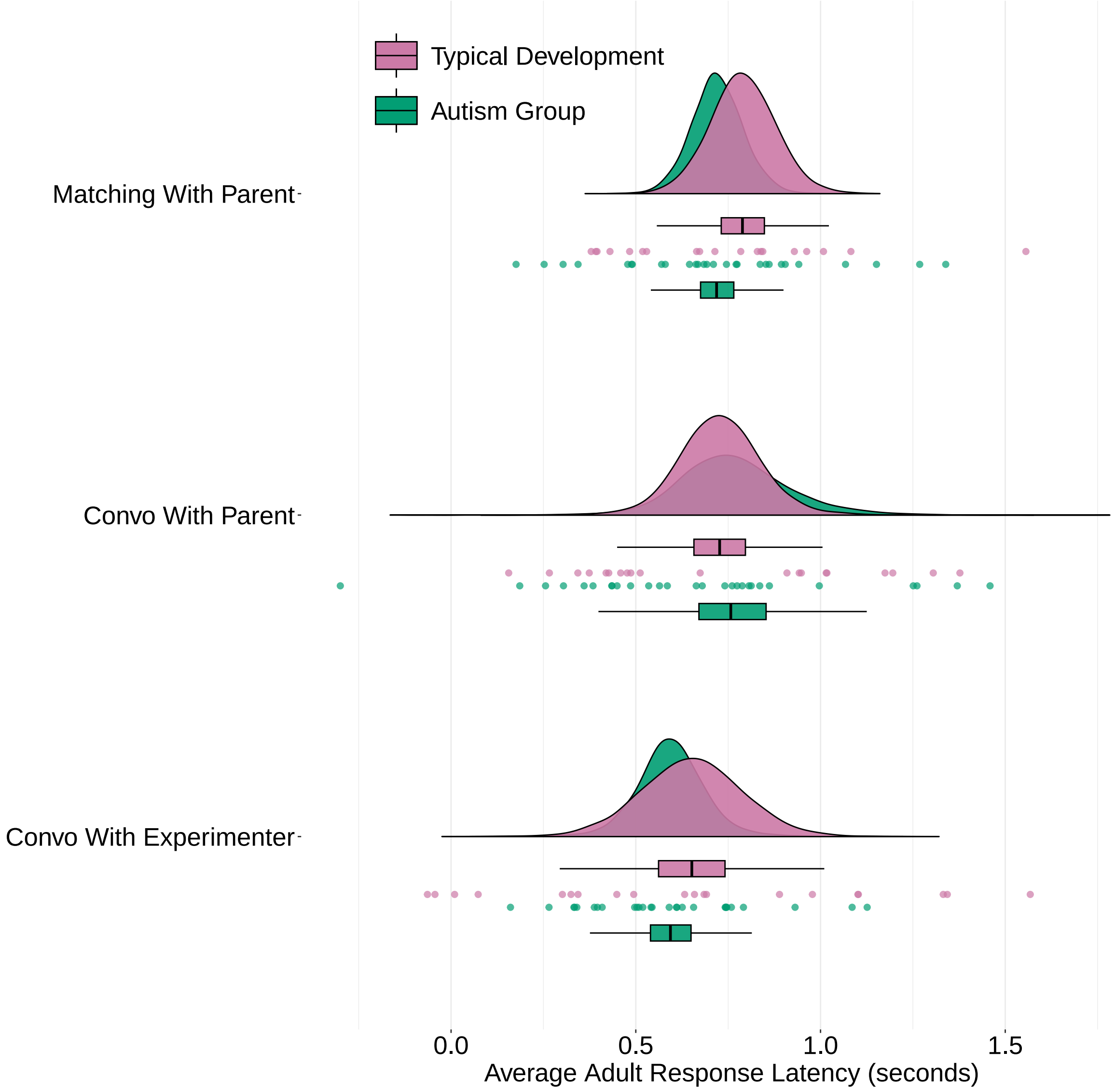

Figure S1: *Model estimates for adult response latencies across different conversational contexts for autistic children (green) and typically developing children (purple). The points represent participant-level posterior predictions from the model. The density plots and boxplots show aggregated posterior predictions for the three different social contexts: Matching Game, Convo With Parent (Conversations with Parents) and Convo With Experimenter (Conversations with Experimenter). The boxplots show the median (vertical line deviding the box), interquartile range (box edges) and most extreme datapoints (whiskers) for the aggregated posterior predictions.*

Table S1: *Posterior estimates for adult response latencies across individual conditions (Matching Game, Convo With Parent (Conversations with Parents) and Convo With Experimenter (Conversations with Experimenter) and aggregated across conditions (Aggregate Estimate) for different model parameters: the Gaussian component (Latency) in milliseconds, proportion of latencies below zero (Overlap Proportion) in proportions, Standard Deviation (Sigma) in milliseconds, exponential component (Beta, or long pauses) on log scale, and Test-Retest Reliability across sessions in correlations.*

	**Tasks**	**Autism Group**	**Typical Development**
**Latency**	Matching With Parent	720ms [606, 837]	790ms [645, 937]
	Convo With Parent	768ms [550, 1031]	728ms [552, 913]
	Convo With Experimenter	595ms [449, 743]	651ms [424, 874]
	Aggregate Estimate	694ms [590, 806]	723ms [612, 834]
**Overlap Proportion**	Matching With Parent	0.3 [0.26, 0.34]	0.26 [0.22, 0.3]
	Convo With Parent	0.22 [0.18, 0.27]	0.22 [0.17, 0.28]
	Convo With Experimenter	0.29 [0.25, 0.33]	0.26 [0.2, 0.33]
	Aggregate Estimate	0.27 [0.24, 0.3]	0.25 [0.22, 0.28]
**Sigma**	Matching With Parent	525ms [487, 566]	469ms [432, 510]
	Convo With Parent	499ms [439, 567]	568ms [492, 658]
	Convo With Experimenter	870ms [812, 933]	785ms [721, 855]
	Aggregate Estimate	611ms [579, 645]	594ms [558, 633]
**Beta**	Matching With Parent	0.19 [0.1, 0.27]	0.15 [0.05, 0.26]
	Convo With Parent	-0.14 [-0.28, 0]	-0.17 [-0.36, 0.01]
	Convo With Experimenter	-0.42 [-0.56, -0.28]	-0.33 [-0.49, -0.18]
	Aggregate Estimate	-0.12 [-0.2, -0.05]	-0.12 [-0.21, -0.02]
**Test-Retest Reliability**	Matching With Parent	0.47 [0.38, 0.56]	0.35 [0.08, 0.53]
	Parent Conversation	0.94 [0.93, 0.95]	0.82 [0.8, 0.86]
	Convo With Experimenter	0.78 [0.74, 0.8]	0.75 [0.72, 0.77]

S.1.1.2 Individual Socio-Cognitive Differencs, Adults

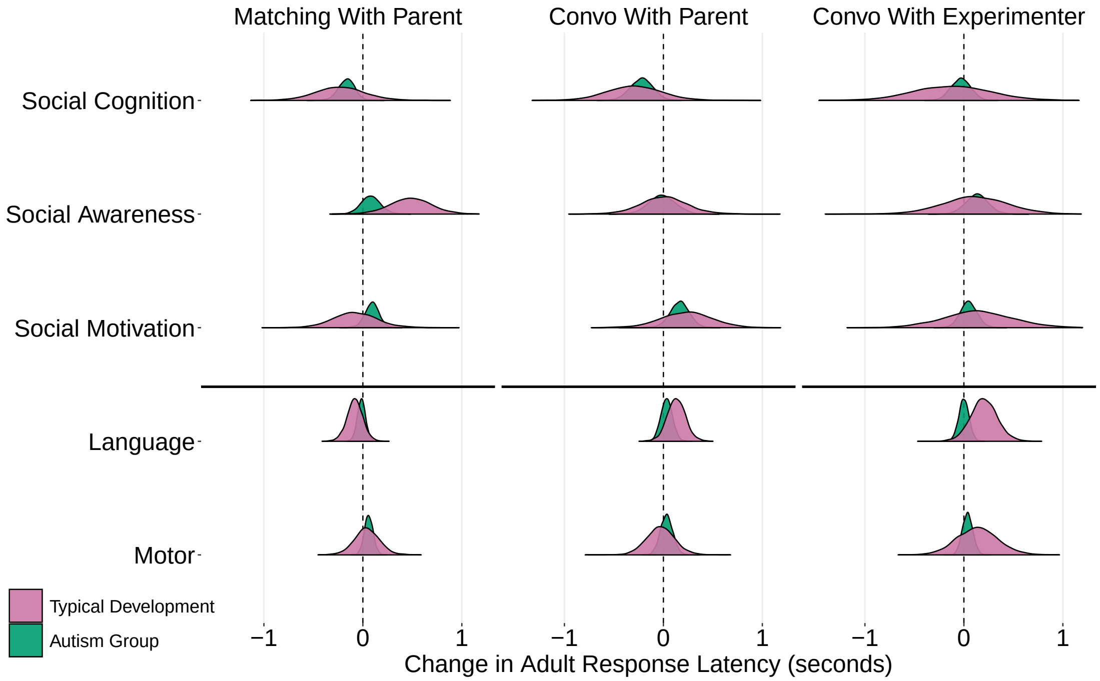

Figure S2: *Model estimates for change in adult response latencies as a function of one standard deviation increase in each measure across the three social contexts: Matching Game, Convo With Parent (Conversations with Parents) and Convo With Experimenter (Conversations with Experimenter). The boxplots show the median (vertical line deviding the box), interquartile range (box edges) and most extreme datapoints (whiskers) for the aggregated posterior predictions. The three top measures (i.e., Social Cognition, Social Awareness, Social Motivation) come from the SRS-2, whereas Language Skills refer to CELF-5 and Motor Skills refer to VAB-3.*

Table S2: *Posterior estimates for how adult response latencies change across individual conditions (Matching Game, Conversations With Parents and Conversations With Experimenter) and all conditions (Aggregate Estimate) as a function of each of the types of skills listed below (Motor, Cognitive Skills, Language Skills, Social Awareness and Social Motivation). The three top measures (i.e., Social Cognition, Social Awareness, Social Motivation) came from the Social Responsiveness Scale, Language skills refer to the Clinical Evaluation of Language Fundamentals, and Motor Skills refer to Vineland Adaptive Behaviour Scales. The estimates denote the change in adult response latency as a function of one standard deviation increase in the skills of the children.*

	**Skills**	**Autism Group**	**Typical Development**
**Social Cognition**	Matching With Parent	-165ms [-302, -33]	-228ms [-639, 192]
	Convo With Parent	-219ms [-407, -37]	-294ms [-751, 167]
	Convo With Experimenter	-31ms [-192, 132]	-118ms [-760, 538]
	Aggregate Estimate	-138ms [-232, -43]	-213ms [-518, 96]
**Social Awareness**	Matching With Parent	81ms [-77, 248]	483ms [127, 833]
	Convo With Parent	-12ms [-231, 213]	6ms [-385, 406]
	Convo With Experimenter	132ms [-55, 313]	112ms [-430, 662]
	Aggregate Estimate	67ms [-46, 182]	201ms [-51, 458]
**Social Motivation**	Matching With Parent	92ms [-29, 209]	-77ms [-437, 296]
	Convo With Parent	164ms [0.82, 323.53]	234ms [-194, 655]
	Convo With Experimenter	49ms [-88, 190]	145ms [-442, 737]
	Aggregate Estimate	102ms [20, 184]	101ms [-169, 378]
**Language**	Matching With Parent	-14ms [-84, 56]	-81ms [-217, 51]
	Convo With Parent	33ms [-63, 130]	131ms [-18, 285]
	Convo With Experimenter	-0.3ms [-86, 85]	204ms [-16, 426]
	Aggregate Estimate	6ms [-44, 57]	85ms [-16, 189]
**Motor**	Matching With Parent	60ms [-13, 133]	41ms [-171, 254]
	Parent Conversations	33ms [-71, 137]	-28ms [-262, 210]
	Convo With Experimenter	37ms [-48, 125]	137ms [-203, 485]
	Aggregate Estimate	43ms [-9, 96]	50ms [-104, 212]

S.1.1.3 Predictability, Adults

Table S3: *Posterior estimates for how adult response latencies change across individual conditions (Matching Game, Parent Conversations and Experimenter Conversations) and all conditions (Aggregate Estimate) as a function of the predictability of the previous child utterance and adult utterance. The estimates denote the change in adult response latency as a function of one standard deviation increase in the predictability of the utterance.*

.

	**Tasks**	**Autism Group**	**Typical Development**
**Predictability of Previous Child Utterance**	Matching With Parent	2.46ms [-26, 32]	37ms [6, 71]
	Convo With Parent	-10ms [-48, 28]	11ms [-29, 52]
	Convo With Experimenter	-36ms [-73, 1]	-34ms [-85, 16]
	Aggregate Estimate	-15ms [-35, 6]	4.66ms [-19, 29]
**Predictability of Own Utterance**	Matching With Parent	-8ms [-29, 14]	-4.16ms [-28, 19]
	Convo With Parent	-14ms [-38, 10]	4.98ms [-25, 35]
	Convo With Experimenter	47ms [14, 80]	55ms [16, 96]
	Aggregate Estimate	8ms [-8, 24]	19ms [-0.35, 37.7]

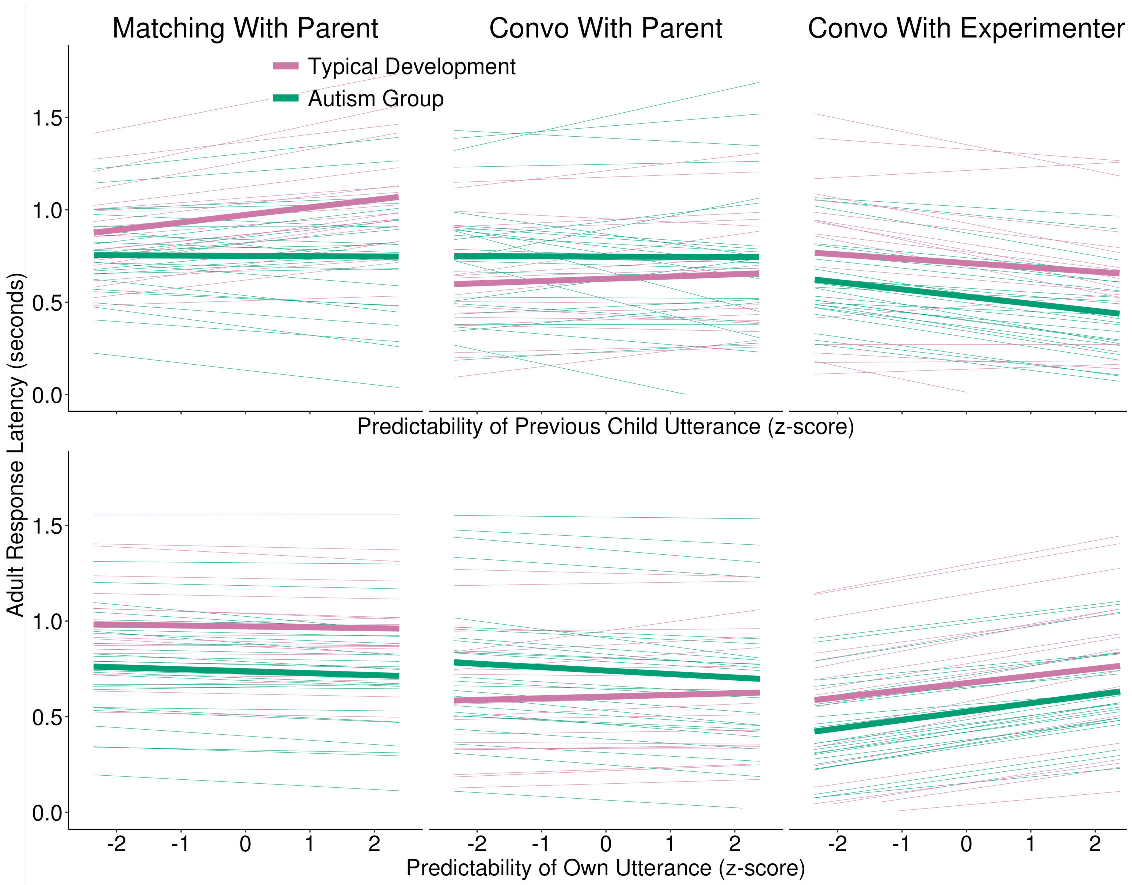

Figure S3: *Model estimates for how adult response latencies change as a function of the predictability of the previous child response latency (top) and predictability of their own utterance (bottom) across the three social contexts: Matching Game, Convo With Parent (Conversations with Parents) and Convo With Experimenter (Conversations with Experimenter). The cosine similarity score here represents scaled values (i.e., one unit increase equals one standard deviation increase in cosine similarity). The faded lines are posterior predictions from the model for individual adult participants in the study, whereas the thicker lines are average predictions.*

S.1.1.4 Shared Tempo, Adults

Table S4: *Posterior estimates for how adult response latencies change across individual conditions (Matching Game, Parent Conversations and Experimenter Conversations) and all conditions (Aggregate Estimate) as a function of the latency of the previous child utterance (Interpersonal Adjustment) and previous adult response latency (Self-Adjustment). The estimates denote the change in adult response latency as a function of a one-second increase in the response latency of the previous utterance.*

	**Tasks**	**Autism Group**	**Typical Development**
**Interpersonal Adjustment**	Matching With Parent	40ms [24, 58]	20ms [-8, 47]
	Convo With Parent	37ms [6, 70]	40ms [-12, 96]
	Convo With Experimenter	27ms [-10, 66]	45ms [0.85, 92.23]
	Aggregate Estimate	35ms [18, 53]	35ms [10, 61]
**Self Adjustment**	Matching With Parent	38ms [21, 55]	32ms [8, 58]
	Convo With Parent	67ms [38, 97]	46ms [10, 82]
	Convo With Experimenter	102ms [57, 148]	84ms [47, 121]
	Aggregate Estimate	69ms [50, 88]	54ms [35, 73]

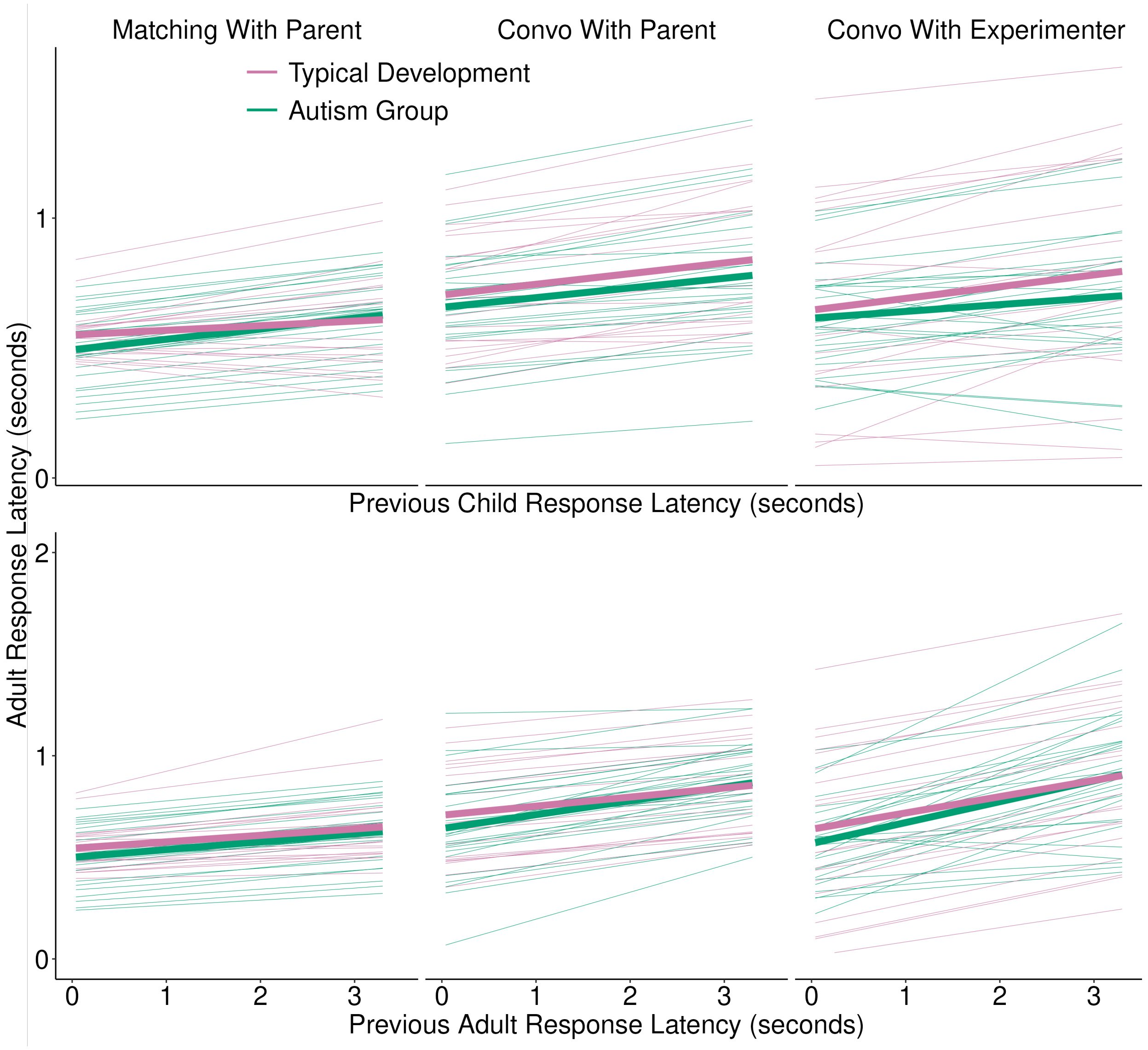

Figure S4: *Model estimates for how adult response latencies change with the latency of the previous child response latency (top) and the previous adult response latency (bottom) across the three social contexts: Matching Game, Convo With Parent (Conversations with Parents) and Convo With Experimenter (Conversations with Experimenter). The faded lines are posterior predictions from the model for individual adult participants in the study, whereas the thicker lines are average predictions.*

S.1.2. Test-Retest Reliability

To assess the consistency of individual children's response latencies across different social contexts, we calculated correlations between posterior estimates of each child's mean latency in each context. Cross-context consistency was quantified as the mean of all pairwise correlations between the three social contexts for each diagnostic group. Bootstrap confidence intervals (n = 1000) were computed to estimate uncertainty around these consistency measures. High cross-context consistency indicates that children who exhibit faster (or slower) response latencies in one social context tend to maintain this relative standing across other contexts, suggesting stable individual differences in conversational timing patterns. Cross-context consistency was moderate to good for both groups (Autism Group: r = 0.65, 95% CI [0.38, 0.82]; Typical Development: r = 0.56, 95% CI [0.21, 0.83]), indicating that individual differences in turn-taking latencies represent relatively stable characteristics of children's conversational behaviour across diverse social situations (see **Figure S5**).

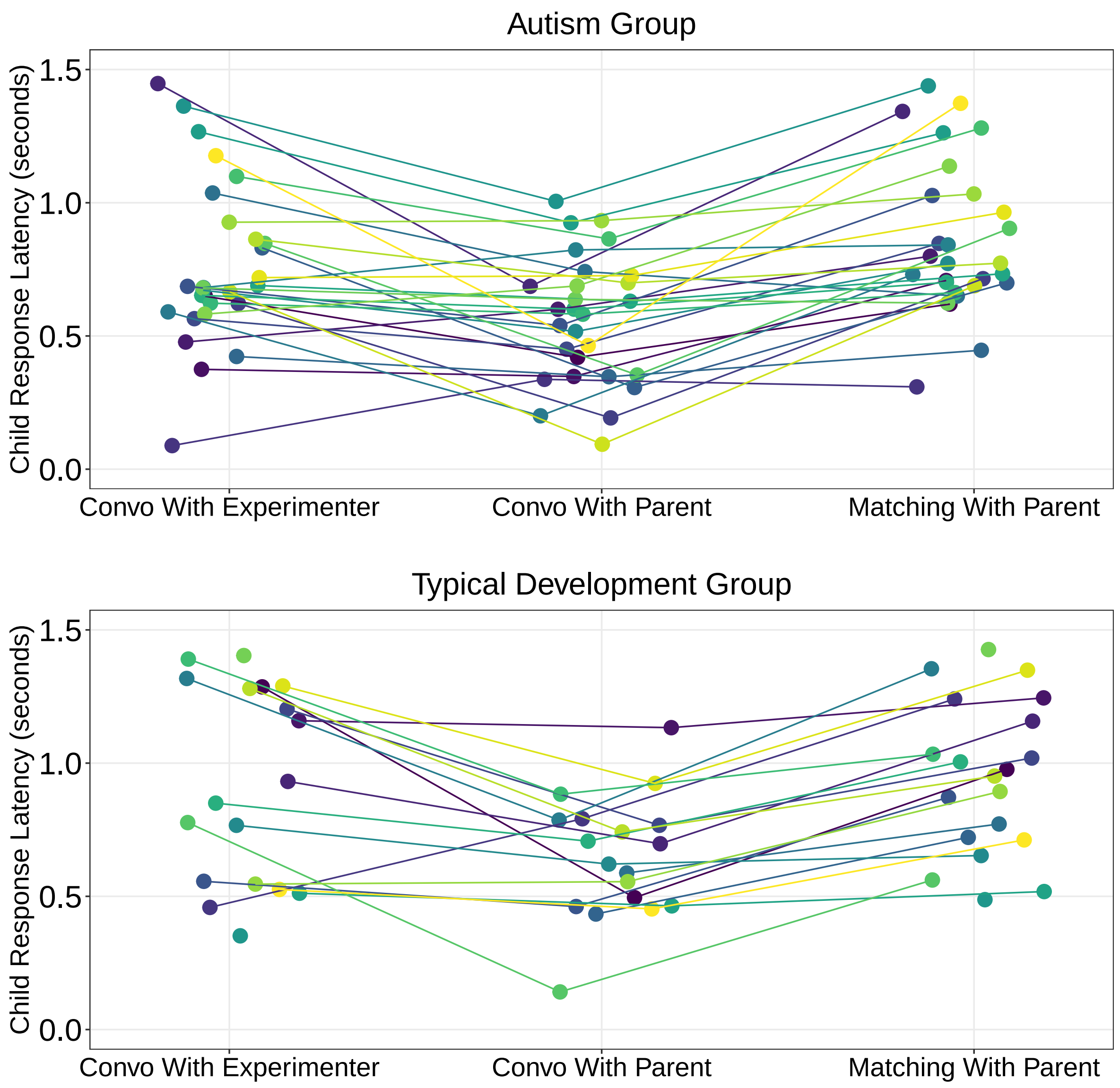

Figure S5: *Panel of plots showing correlations among estimates of child-specific response latencies across different social contexts. Each point is the model estimate for a specific child and each child is connected across contexts with a line.*

S.1.3. Control Analyses

S.1.3.1 Child Latency With No Overlaps

To check the extent to which the inclusion of overlaps influenced our estimates in the main manuscript, we ran control model without overlaps, the estimates for which are in **Table S5**.

Table S5: *Posterior estimates for child response latencies across individual conditions (Matching Game, Convo With Parent (Conversations with Parents) and Convo With Experimenter (Conversations with Experimenter) and aggregated across conditions (Aggregate Estimate) for different model parameters: the Gaussian component (Latency) in milliseconds, Standard Deviation (Sigma) in milliseconds, and exponential component (Beta, or long pauses) on log scale.*

	**Tasks**	**Autism Group**	**Typical Development**
**Latency**	Matching With Parent	1238ms [1181, 1298]	1328ms [1246, 1414]
	Convo With Parent	873ms [808, 938]	885ms [788, 986]
	Convo With Experimenter	1265ms [1165, 1360]	1454ms [1327, 1581]
	Aggregate Estimate	1125ms [1077, 1174]	1222ms [1152, 1291]
**Beta**	Matching With Parent	0.2 [0.15, 0.25]	0.27 [0.2, 0.33]
	Convo With Parent	-0.17 [-0.24, -0.09]	-0.17 [-0.27, -0.06]
	Convo With Experimenter	-0.09 [-0.17, -0.01]	-0.07 [-0.19, 0.05]
	Aggregate Estimate	-0.02 [-0.06, 0.03]	0.01 [-0.05, 0.07]
**Sigma**	Matching With Parent	7ms [3.76, 11.67]	6ms [3.33, 10.76]
	Convo With Parent	10ms [4.82, 20.03]	23ms [10, 45]
	Convo With Experimenter	201ms [163, 245]	326ms [286, 372]
	Aggregate Estimate	24ms [18, 33]	36ms [25, 48]

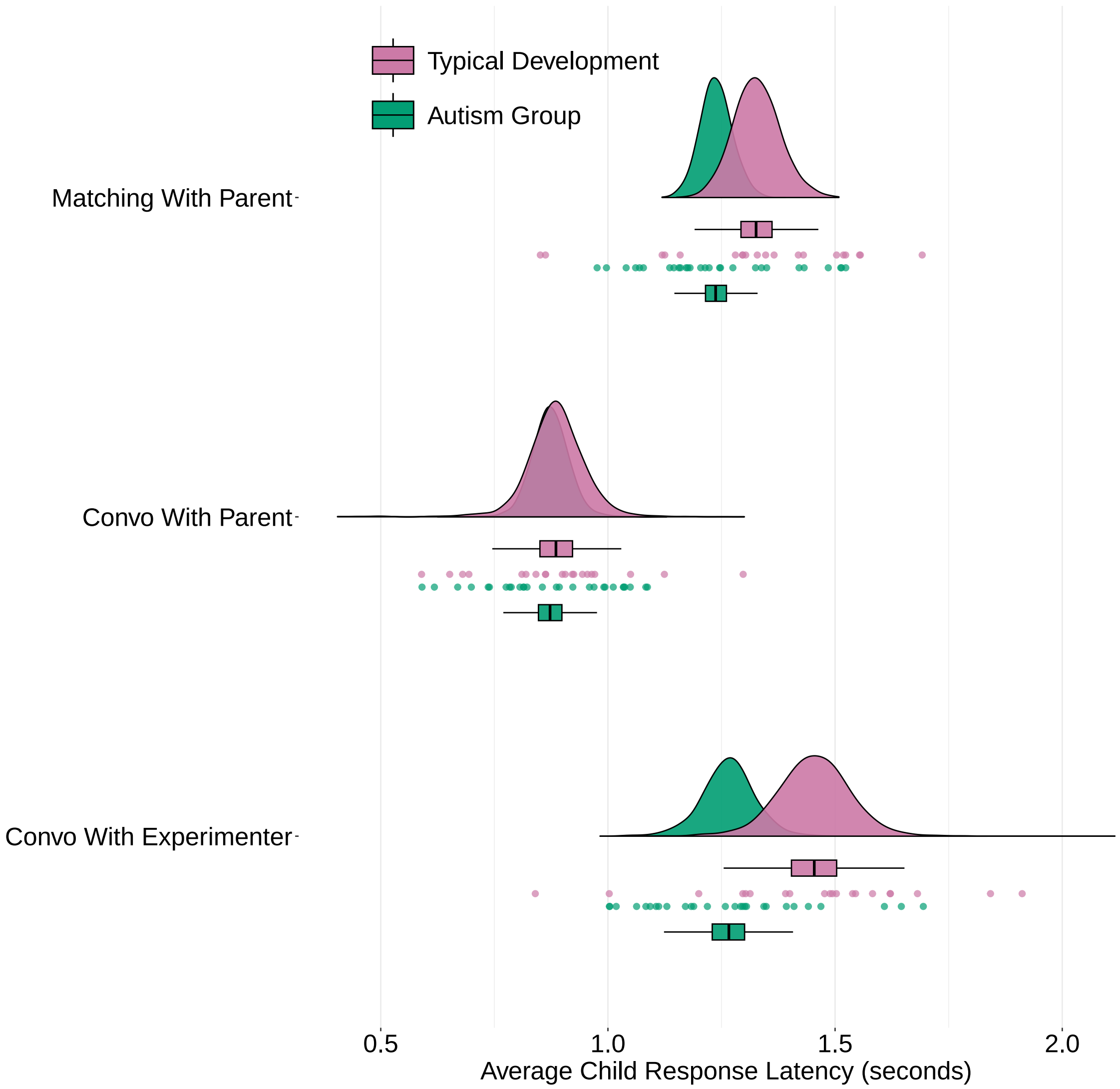

Figure S6: *Model estimates for child response latencies across different conversational contexts for autistic children (green) and typically developing children (purple) when excluding overlaps from the model. The points represent participant-level posterior predictions from the model. The density plots and boxplots show aggregated posterior predictions for the three different social contexts: Matching Game, Convo With Parent (Conversations with Parents) and Convo With Experimenter (Conversations with Experimenter). The boxplots show the median (vertical line deviding the box), interquartile range (box edges) and most extreme datapoints (whiskers) for the aggregated posterior predictions.*

S.1.3.2 Adult Latency With No Overlaps

Table S6: *Posterior estimates for adult response latencies across individual conditions (Matching Game, Convo With Parent (Conversations with Parents) and Convo With Experimenter (Conversations with Experimenter) and aggregated across conditions (Aggregate Estimate) for different model parameters: the Gaussian component (Latency) in milliseconds, Standard Deviation (Sigma) in milliseconds, and exponential component (Beta, or long pauses) on log scale.*

	**Tasks**	**Autism Group**	**Typical Development**
**Latency**	Matching With Parent	1195ms [1135, 1257]	1192ms [1092, 1298]
	Convo With Parent	942ms [853, 1027]	1021ms [898, 1157]
	Convo With Experimenter	1135ms [1032, 1240]	1144ms [1009, 1279]
	Aggregate Estimate	1090ms [1036, 1142]	1119ms [1046, 1196]
**Beta**	Matching With Parent	0.17 [0.12, 0.22]	0.14 [0.05, 0.22]
	Convo With Parent	-0.09 [-0.16, -0.01]	-0.06 [-0.18, 0.07]
	Convo With Experimenter	-0.34 [-0.47, -0.21]	-0.31 [-0.43, -0.19]
	Aggregate Estimate	-0.08 [-0.14, -0.03]	-0.07 [-0.14, -0.01]
**Sigma**	Matching With Parent	6ms [3.1, 9.5]	7ms [3.73, 11.57]
	Convo With Parent	9ms [4.62, 14.61]	15ms [9, 26]
	Convo With Experimenter	249ms [217, 284]	234ms [197, 276]
	Aggregate Estimate	23ms [17, 30]	29ms [22, 37]

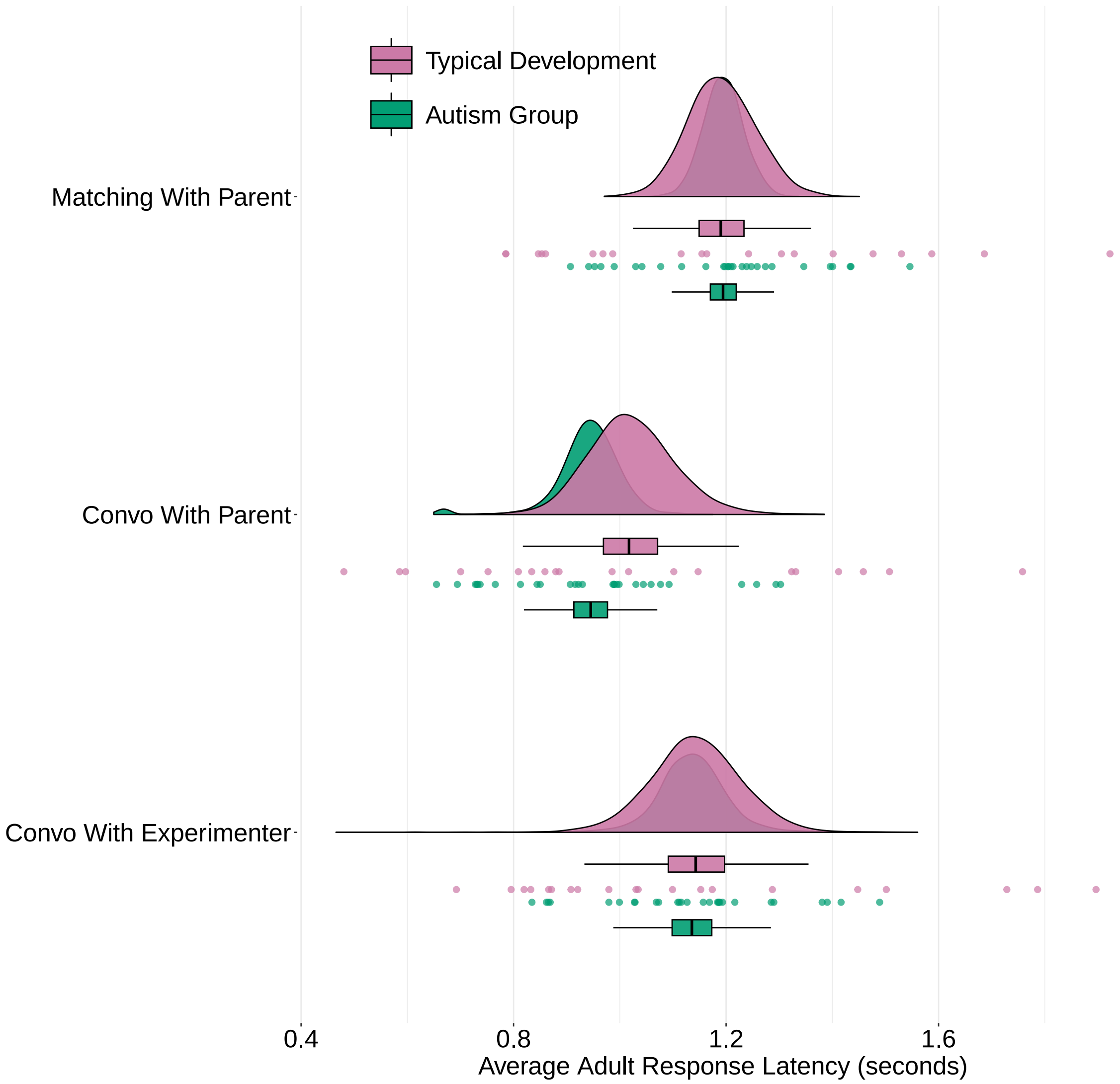

Figure S7: *Model estimates for adult response latencies across different conversational contexts for autistic children (green) and typically developing children (purple) when excluding overlaps from the model. The points represent participant-level posterior predictions from the model. The density plots and boxplots show aggregated posterior predictions for the three different social contexts: Matching Game, Convo With Parent (Conversations with Parents) and Convo With Experimenter (Conversations with Experimenter). The boxplots show the median (vertical line deviding the box), interquartile range (box edges) and most extreme datapoints (whiskers) for the aggregated posterior predictions*

S.1.3.3 Child Latency Across Gender

Table S7: *Posterior estimates for child response latencies across genders and individual conditions (Matching Game, Convo With Parent (Conversations with Parents) and Convo With Experimenter (Conversations with Experimenter) and aggregated across conditions (Aggregate Estimate).*

	**Male**	**Female**	**NonBinary**
**Autism Group**			
Matching With Parent	892ms [755, 1029]	830ms [679, 981]	740ms [428, 1041]
Convo With Parent	621ms [496, 748]	521ms [356, 690]	412ms [70, 773]
Convo With Experimenter	793ms [646, 938]	738ms [552, 926]	696ms [286, 1114]
Aggregate	769ms [672, 864]	697ms [577, 817]	616ms [357, 883]
**Typical Development**			
Matching With Parent	1033ms [853, 1213]	895ms [748, 1040]	
Convo With Parent	719ms [505, 934]	626ms [454, 799]	
Convo With Experimenter	941ms [670, 1208]	1061ms [829, 1282]	
Aggregate	898ms [743, 1051]	860ms [737, 982]	

S.1.3.4 Child Latency With No Sigma and No Beta

To explore how the estimates change with varying beta and sigma components, we ran a control model without the varying beta and sigma components. The posterior estimates from this model can be viewed in **Table S8** below.

Table S8: *Posterior estimates for child response latencies across individual conditions (Matching Game, Convo With Parent (Conversations with Parents) and Convo With Experimenter (Conversations with Experimenter) and aggregated across conditions (Aggregate Estimate) for different model parameters without a varying beta and sigma. SD refers to standard deviation of intercepts according to child and visit.*

	**Tasks**	**Autism Group**	**Typical Development**
**Latency**	Matching With Parent	785ms [704, 868]	813ms [729, 899]
	Convo With Parent	726ms [585, 867]	832ms [662, 1002]
	Convo With Experimenter	766ms [629, 909]	970ms [761, 1177]
	Aggregate Estimate	759ms [672, 844]	872ms [764, 981]
**Child SD**	Matching With Parent	204ms [157, 261]	195ms [137, 269]
	Convo With Parent	210ms [157, 277]	340ms [253, 447]
	Convo With Experimenter	352ms [277, 442]	460ms [354, 589]
	Aggregate Estimate	256ms [215, 303]	332ms [276, 395]
**Visit SD**	Matching With Parent	69ms [25, 134]	42ms [3.62, 100.02]
	Convo With Parent	115ms [16, 308]	78ms [4.7, 254.14]
	Convo With Experimenter	81ms [8, 214]	114ms [26, 273]
	Aggregate Estimate	88ms [37, 172]	78ms [30, 158]

S.1.3.5 Child Latency With Sigma But No Beta

To explore how the estimates change with a vaying beta component, we ran a control model without the varying beta component but retained the varying sigma component. The posterior estimates from this model can be viewed in **Table S9** below.

Table S9: *Posterior estimates for child response latencies across individual conditions (Matching Game, Convo With Parent (Conversations with Parents) and Convo With Experimenter (Conversations with Experimenter) and aggregated across conditions (Aggregate Estimate) for different model parameters with a varying sigma but no beta.*

	**Tasks**	**Autism Group**	**Typical Development**
**Latency**	Matching With Parent	758ms [679, 839]	786ms [711, 868]
	Convo With Parent	699ms [584, 809]	793ms [642, 947]
	Convo With Experimenter	830ms [699, 957]	1057ms [855, 1256]
	Aggregate Estimate	762ms [683, 837]	879ms [779, 982]
**Sigma**	Matching With Parent	645ms [599, 694]	569ms [520, 622]
	Convo With Parent	495ms [440, 557]	571ms [496, 660]
	Convo With Experimenter	897ms [834, 963]	879ms [808, 956]
	Aggregate Estimate	659ms [626, 695]	659ms [617, 701]
**Child SD**	Matching With Parent	189ms [145, 242]	183ms [130, 251]
	Convo With Parent	204ms [151, 267]	308ms [233, 404]
	Convo With Experimenter	328ms [259, 410]	453ms [340, 592]
	Aggregate Estimate	240ms [202, 282]	315ms [260, 377]
**Visit SD**	Matching With Parent	72ms [31, 134]	38ms [2.98, 93.19]
	Convo With Parent	82ms [6, 248]	80ms [4.95, 248.07]
	Convo With Experimenter	84ms [11, 213]	94ms [9, 240]
	Aggregate Estimate	79ms [34, 153]	71ms [23, 147]

S.1.3.6 Child Overlaps according to Individual Differences

To explore the extent to which potential increases or decreases in response latencies were a function of a greater proportion of overlaps, we modelled the proportion of overlaps (i.e., negative latencies) according to individual differences among children, as shown in **Figure S8**.

The Bayesian logistic regression with a Bernoulli likelihood revealed several credible individual difference effects on overlap probability. Most notably, cognitive skills showed a strong positive association with overlaps for typically developing children in conversations with their parents (β = 1.13 [0.08, 2.14], ER = 52). A smaller but credible positive effect of cognitive skills was also observed for children with ASD during familiar question tasks (β = 0.42 [0.00, 0.85], ER = 40). These findings suggest that children with better cognitive abilities were substantially more likely to produce overlapping speech during familiar question contexts.

In contrast, motivation showed diverging effects between diagnostic groups. For children with autism, higher motivation was associated with reduced overlap probability in conversations with parents (β = -0.27 [-0.57, 0.03], ER = 14) and the matching game (β = -0.26 [-0.51, -0.01], ER = 22), while the effects for typically developing children were positive but less certain (e.g., for matching game: β = 0.31 [-0.21, 0.84], ER = 5). This pattern suggests that motivation may lead to more controlled responding in children with autism but potentially faster, more overlapping responses in typically developing children.

Additionally, awareness skills showed a credible negative effect for typically developing children during unfamiliar question tasks (β = -0.70 [-1.53, 0.16], ER = 18), suggesting that higher awareness may lead to more controlled responding that reduces overlaps in challenging contexts. Other individual difference measures, including language, and motor skills, showed weaker or non-credible associations with overlap probability, with most 95% credible intervals including zero. Overall, cognitive skills emerged as the strongest predictor of overlap behaviour, particularly for typically developing children in familiar conversational

contexts.

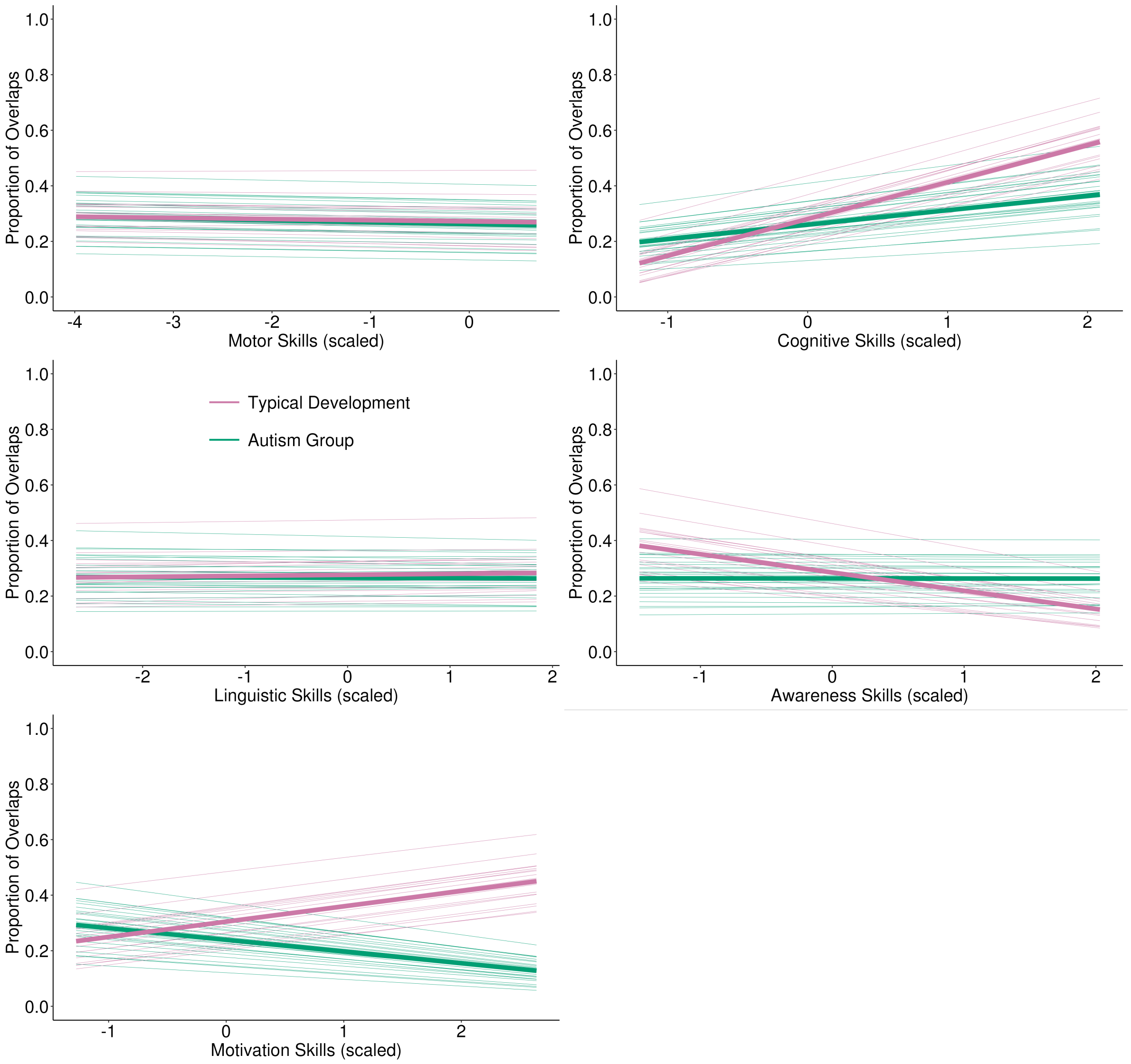

Figure S8: *This panel of plots displays the proportion of overlaps according to individual differences of the children. The faded lines are posterior predictions from model for individual subjects and how the proportion of overlaps changes for each of the continuous predictors.*

S.1.3.7 Child Individual Differences With No Overlaps

To check the extent to which overlaps were driving the estimates of change in response latencies according to individual differences, we ran the same individual differences model on data where overlaps (i.e., negative latencies) were excluded. The posterior estimates from this model can be viewed in **Table S10** below.

Table S10: *Posterior estimates for models when excluding overlaps for how child response latencies change across individual conditions (Matching Game, Parent Conversations and Experimenter Conversations) and all conditions (Aggregate Estimate) as a function of each of the types of skills listed below (Motor, Cognitive Skills, Language Skills, Social Awareness and Social Motivation). The estimates denote the change in child response latency as a function of one standard deviation increase in the skills of the children.*

	**Skills**	**Autism Group**	**Typical Development**
**Social Cognition**	Matching With Parent	-13ms [-42, 15]	-95ms [-382, 147]
	Convo With Parent	12ms [-30, 55]	50ms [-171, 291]
	Convo With Experimenter	-76ms [-176, 27]	-490ms [-883, -83]
	Aggregate Estimate	-26ms [-67, 16]	-178ms [-367, -4]
**Social Awareness**	Matching With Parent	4.24ms [-29, 37]	69ms [-81, 252]
	Convo With Parent	27ms [-21, 77]	-59ms [-205, 81]
	Convo With Experimenter	-4.11ms [-117, 108]	190ms [-65, 449]
	Aggregate Estimate	9ms [-39, 56]	67ms [-44, 189]
**Social Motivation**	Matching With Parent	4.48ms [-21, 30]	-46ms [-219, 116]
	Convo With Parent	-26ms [-64, 11]	78ms [-77, 245]
	Convo With Experimenter	84ms [-3.38, 169.97]	90ms [-194, 377]
	Aggregate Estimate	21ms [-15, 57]	40ms [-84, 165]
**Language**	Matching With Parent	1.94ms [-14, 18]	20ms [-33, 84]
	Convo With Parent	16ms [-8, 43]	-18ms [-73, 35]
	Convo With Experimenter	0.01ms [-57, 58]	35ms [-59, 131]
	Aggregate Estimate	6ms [-18, 31]	12ms [-29, 55]
**Motor**	Matching With Parent	0.18ms [-18, 18]	11ms [-69, 94]
	Convo With Parent	-6ms [-35, 21]	-9ms [-89, 67]
	Convo With Experimenter	41ms [-21, 101]	-15ms [-155, 130]
	Aggregate Estimate	12ms [-14, 36]	-4.13ms [-67, 57]

S.1.3.8 Predictability

As shown in **Table S11**, the overall degree of utterance predictability differed slightly across interactional contexts, with both the child and adult utterances agreeing with each other in terms of their overall degree of predictability.

Table S11: *Overview of Average Utterance Predictability Across Different Contexts*

Condition	Diagnosis	Adult Predictability	Child Predictability
Matching With Parent	Autism Group	0 (0.98)	-0.02 (0.96)
Matching With Parent	Typical Development	-0.02 (1.02)	-0.07 (0.97)
Convo With Parent	Autism Group	0.08 (1.05)	0.19 (1.02)
Convo With Parent	Typical Development	0.08 (1.1)	0.23 (1.04)
Convo With Experimenter	Autism Group	-0.08 (1)	0.01 (0.98)
Convo With Experimenter	Typical Development	-0.15 (0.97)	-0.03 (0.98)

S.1.3.9 Child Latency With Predictability, No Short Utterances

To check the potential influence of shorter utterances on the posterior estimates, we removed all utterances under three words in the corpus and ran the same predictability model as in the main manuscript. The estimates from this control model are shown in **Table S12**.

Table S12: *Posterior estimates for how child response latencies change across individual conditions (Matching With Parent, Parent Conversations and Experimenter Conversations) and all conditions (Aggregate Estimate) as a function of the predictability of the previous adult utterance and child utterance. The estimates denote the change in child response latency as a function of one standard deviation increase in the predictability of the utterance (i.e., cosine similarity).*

	Tasks	Autism Group	Typical Development
**Predictability of Previous Adult Utterance**	Matching With Parent	-54ms [-93, -14]	33ms [-18, 86]
	Convo With Parent	-25ms [-66, 17]	-72ms [-136, -6]
	Convo With Experimenter	-36ms [-93, 21]	-11ms [-83, 61]
	Aggregate Estimate	-38ms [-66, -11]	-17ms [-53, 20]
**Predictability of Own Utterance**	Matching With Parent	-31ms [-60, -3]	-7ms [-47, 32]
	Convo With Parent	10ms [-26, 47]	-12ms [-58, 34]
	Convo With Experimenter	188ms [145, 230]	108ms [50, 166]
	Aggregate Estimate	56ms [34, 77]	29ms [1.13, 56.88]

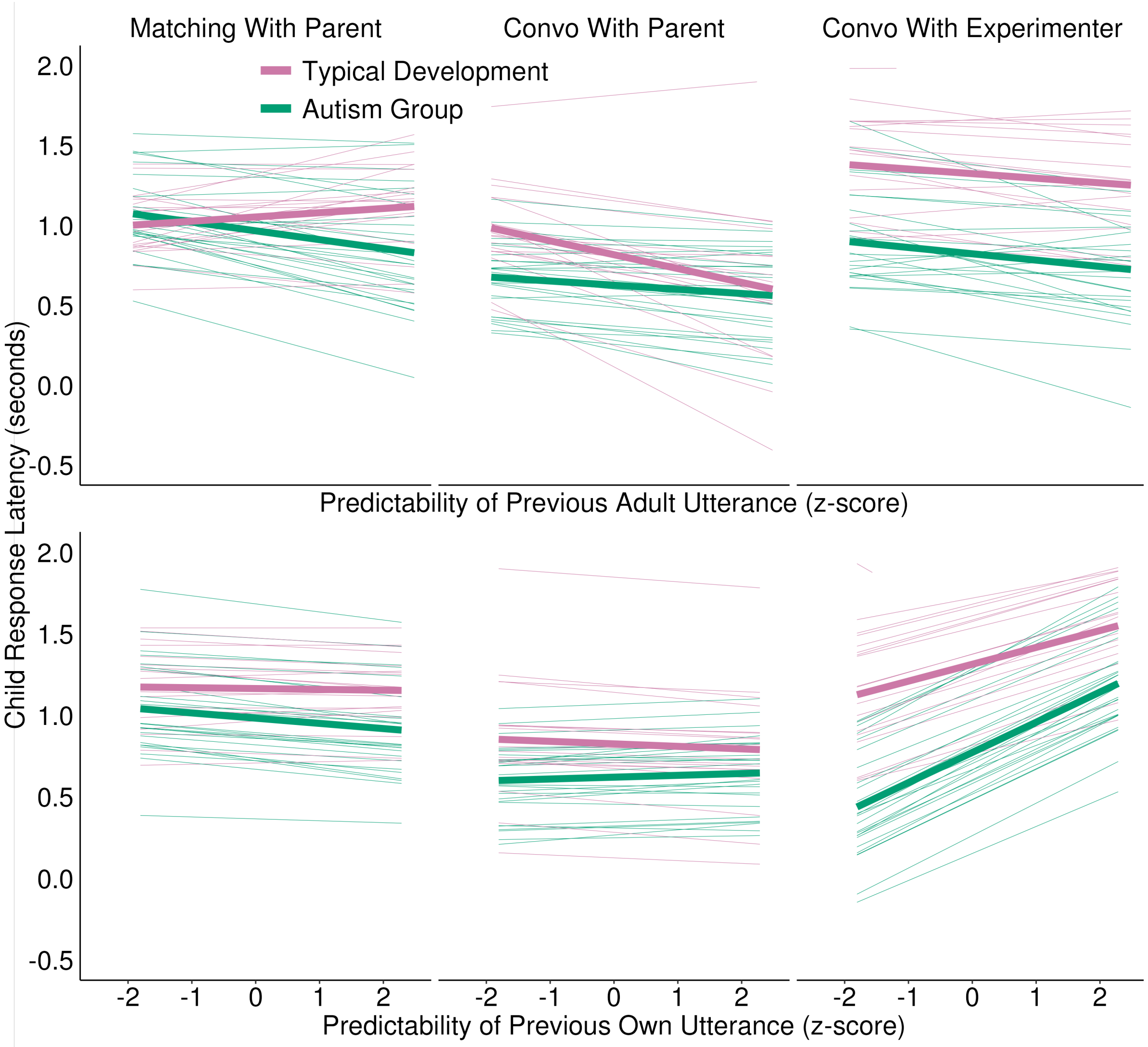

Figure S9: *Posterior estimates in a model excluding utterances below three words. The plot shows how child response latencies change across individual conditions (Matching Game, Parent Conversations and Experimenter Conversations) and all conditions (Aggregate Estimate) as a function of the predictability of the previous adult utterance and current child utterance.*

S.1.3.10 Child Latency With Increasing Familiarity

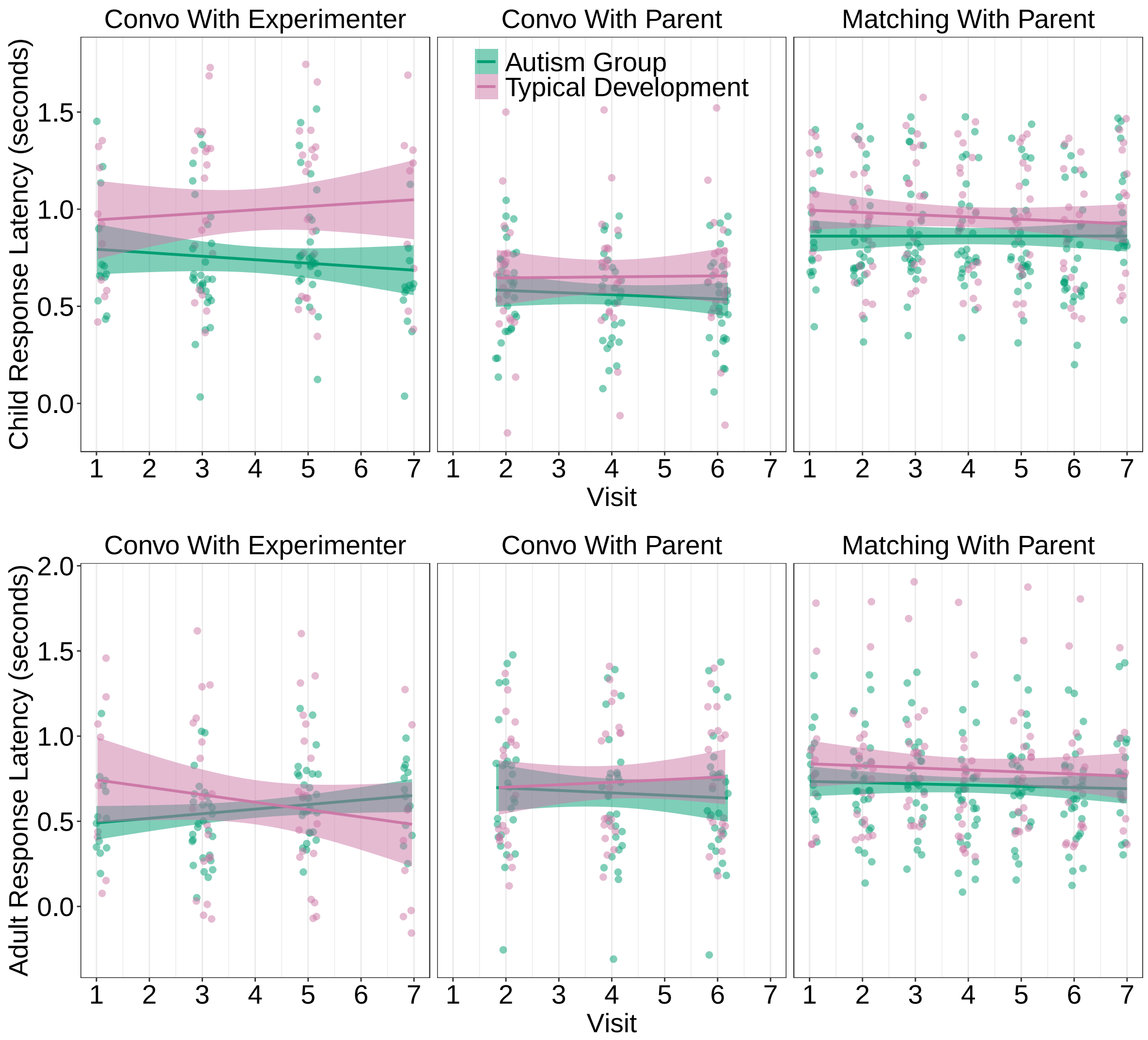

Figure S10: *Posterior estimates for child response latencies across individual conditions (Matching Game, Parent Conversations and Experimenter Conversations) as a function of visit. Individual points denote participant-level posterior estimates from the model for the children (top) and adults (bottom). Note that conversations took place alternately with caregivers (in even weeks) and the experimenter (in odd weeks).*

S.1.3.11 Child Latency For Surrogate Pairs

As a control analysis, we ran the first model after rotating the dataset; that is, pairing the utterances of one child with the interactant of another child and recalculating the response latencies between turns. The estimates from this control model are shown in **Table S13**.

Table S13: *Posterior estimates for child response latencies across individual conditions (Matching Game, Convo With Parent and Experimenter Conversations) and aggregated across conditions (Aggregate Estimate) for different model parameters: the Gaussian component (Latency), exponential component (Beta), Residual Heterogeneity (Sigma), between-subjects standard deviation (Child SD), standard deviation across visits (Visit SD), Residual Heterogeneity across visits (Sigma Visit) and proportion of latencies below zero (Overlap Proportion).*

	**Tasks**	**Autism Group**	**Typical Development**
**Latency**	Matching With Parent	27ms [-1632, 1806]	-202ms [-1896, 1544]
	Convo With Parent	-1823ms [-2576, -1087]	-2070ms [-3003, -1079]
	Convo With Experimenter	-2927ms [-3751, -2156]	-2009ms [-2671, -1330]
	Aggregate Estimate	-1574ms [-2293, -831]	-1427ms [-2183, -632]
**Sigma**	Matching With Parent	17735ms [13366, 23464]	16909ms [12333, 23217]
	Convo With Parent	4737ms [3299, 6966]	4662ms [2355, 8587]
	Convo With Experimenter	8091ms [5725, 11433]	5827ms [3685, 9164]
	Aggregate Estimate	8793ms [7070, 10988]	7716ms [5553, 10557]
**Child SD**	Matching With Parent	3015ms [1756, 4627]	3100ms [1917, 4587]
	Convo With Parent	1102ms [744, 1526]	823ms [458, 1261]
	Convo With Experimenter	1579ms [1070, 2196]	1096ms [687, 1611]
	Aggregate Estimate	1899ms [1405, 2496]	1673ms [1224, 2220]

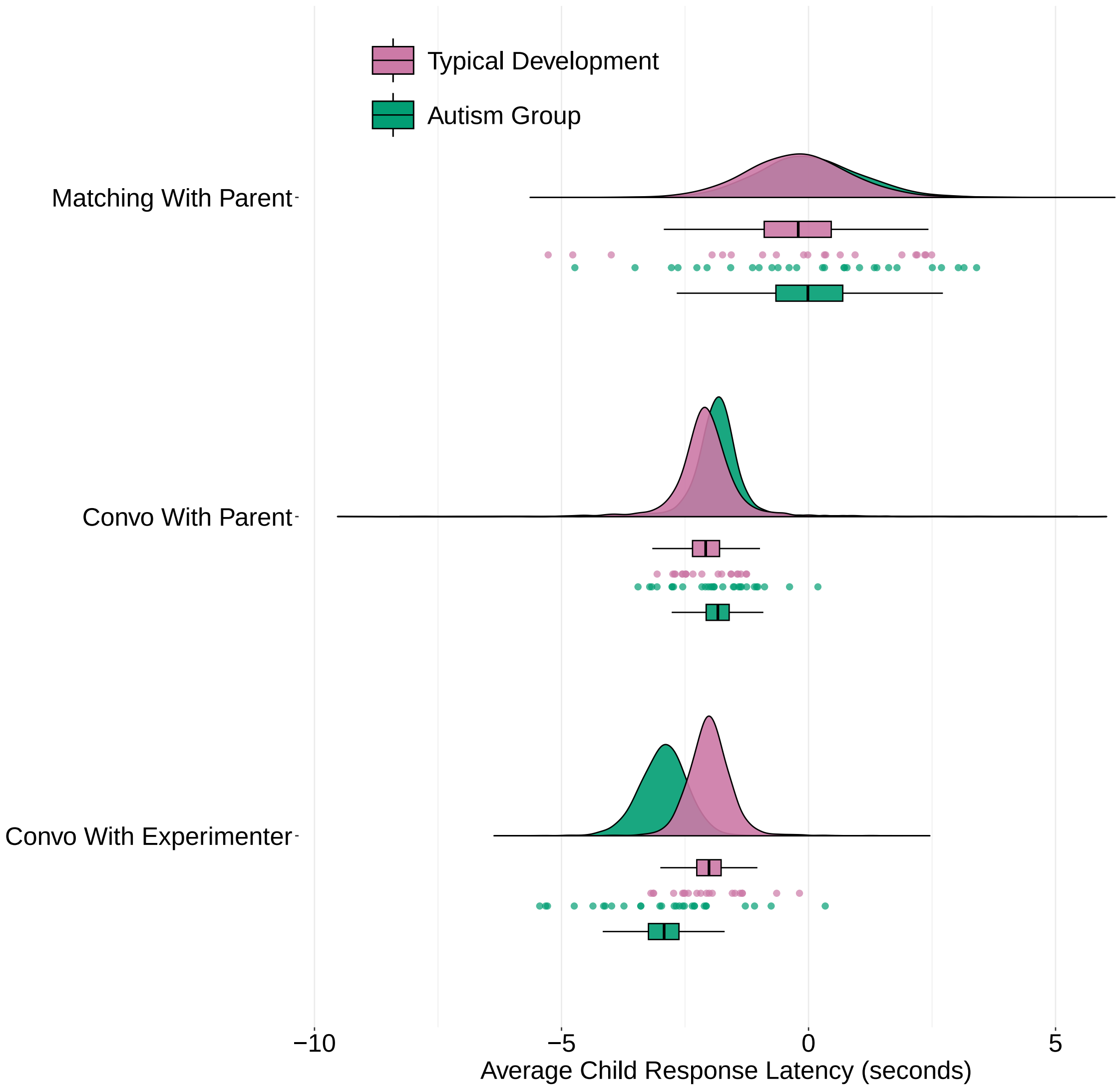

Figure S11: *Posterior estimates for surrogate child response latencies across individual conditions (Matching Game, Parent Conversations and Experimenter Conversations).*

S.1.4. Model Information and Quality Checks

S.1.4.1 Choice of Priors

We chose weakly informative priors in order to ensure that their influence on the estimates was small and to discount extreme effect sizes as unlikely (Lemoine, 2019; Gelman et al., 2017). For the overall distribution, we chose an ex-Gaussian distribution. Our prior for the Gaussian component was specified as having a mean of 1 and standard deviation of 1 based on our prior expectations for child response latencies in a turn-taking context (Nguyen et al., 2022). This prior implies that we expect approximately 95% of the child latencies to fall between -1sec and 3sec. For the between-subject variability across different conversational contexts, we encoded our expectations with a truncated (i.e., standard deviation must have positive values) Gaussian prior with a mean of 0 and a standard deviation of 0.3, which implies that we expect the vast majority of values for the between-subject variability within diagnostic group to be between 0 and 0.6sec. For the rate parameter of the exponential component of the distribution, we specified a Gaussian prior with a mean of 0 and standard deviation of 1 and allowed this to vary for each task and familiarity for each participant.

The models were fitted with Hamiltonian Monte Carlo samplers with 2 parallel chains with 5,000 iterations each, an adapt delta of 0.99 and a maximum tree depth of 20 in order to ensure no divergence in the estimation process. The quality of the models was assessed by (i) ensuring Rhat statistics to be lower than 1.1, (ii) carrying out prior and posterior predictive checks, (iii) plotting prior against posterior estimates and assessing whether the posteriors had lower variance than the priors, (iv) ensuring no divergences in the process of estimation, (v) checking that the number of effective bulk and tail samples was above 200, (vi) conducting prior sensitivity analyses.

All models were fitted using the R package *brms* (Bürkner, 2017). As an example of what the basic *brms* formula looked like, here is the formula for the first model (cf. code on OSF for the full code and *brms* formulae for other models). Note that visit was excluded

from fixed effects for the location parameter as temporal changes in mean response were adequately modeled through random slope structures, while visit-specific heteroscedasticity was explicitly parameterized through fixed effects in the scale parameter (i.e., sigma):

Latency $\sim$ 0 + Diagnosis:Task:Familiarity + (0 + Task:Familiarity | p | gr(ID, by = Diagnosis)) + (0 + Diagnosis:Task:Familiarity | r | Visit),

Sigma $\sim$ 0 + Diagnosis:Task:Familiarity + Diagnosis:Task:Familiarity:Visit + (0 + Task:Familiarity:Visit | p | gr(ID, by = Diagnosis)),

Beta $\sim$ 0 + Diagnosis:Task:Familiarity + (0 + Task:Familiarity | p | gr(ID, by = Diagnosis)) + (0 + Diagnosis:Task:Familiarity | r | Visit)

S.1.4.2 Prior and Posterior Predictive Checks

We performed quality checks of the models by carrying out prior and posterior predictive checks. The below prior predictive checks (on the left) indicate that our priors predict values within the order of magnitude of the distribution. The posterior predictive checks (on the right) indicate that the models have captured the distributions of data. These plots provide reassurance that our models capture relevant aspects of the overall distributions of dependent variables.

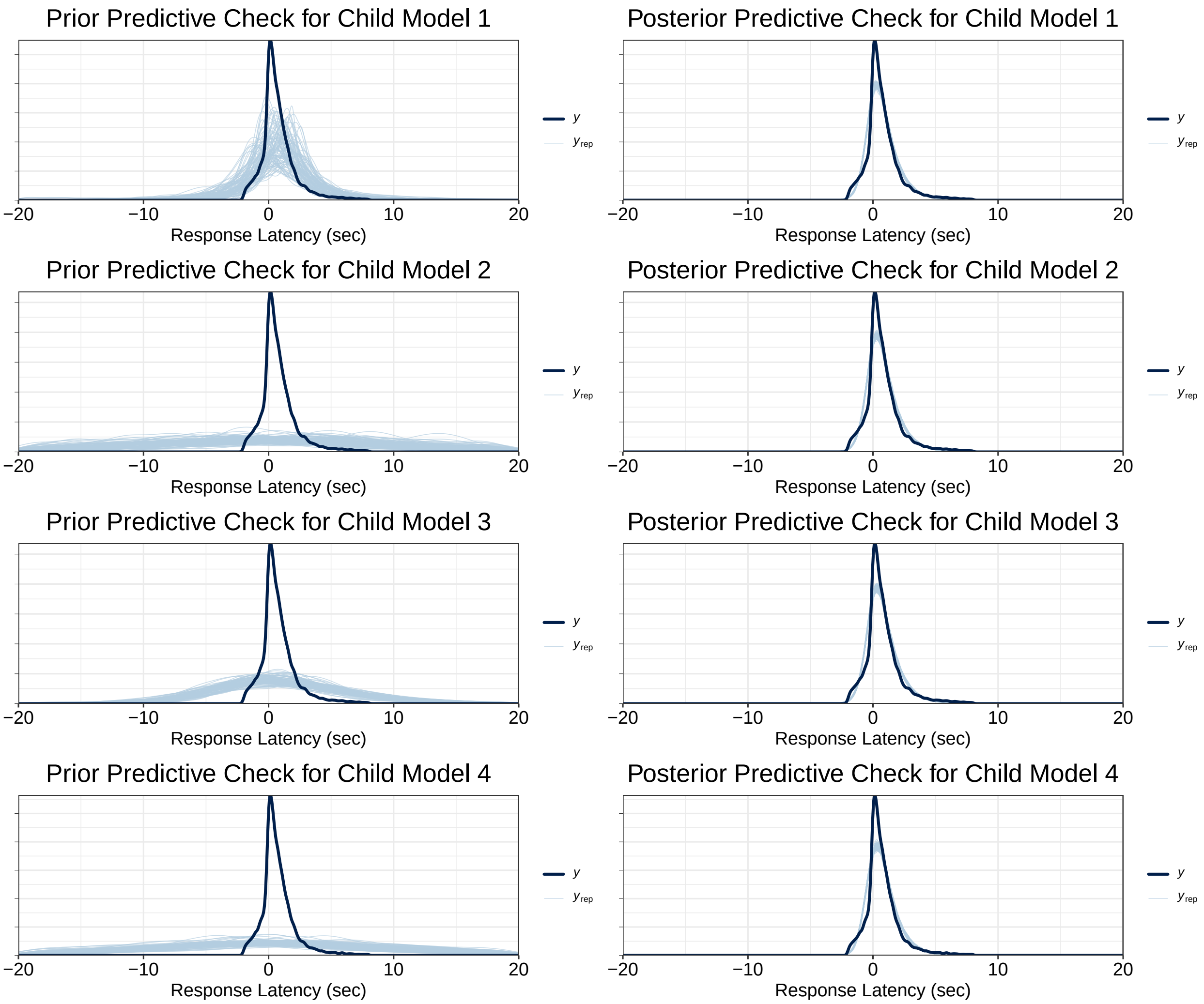

Figure S12: *Prior predictive checks (left column) and posterior predictive checks (right column) for child latency in models 1-4.*

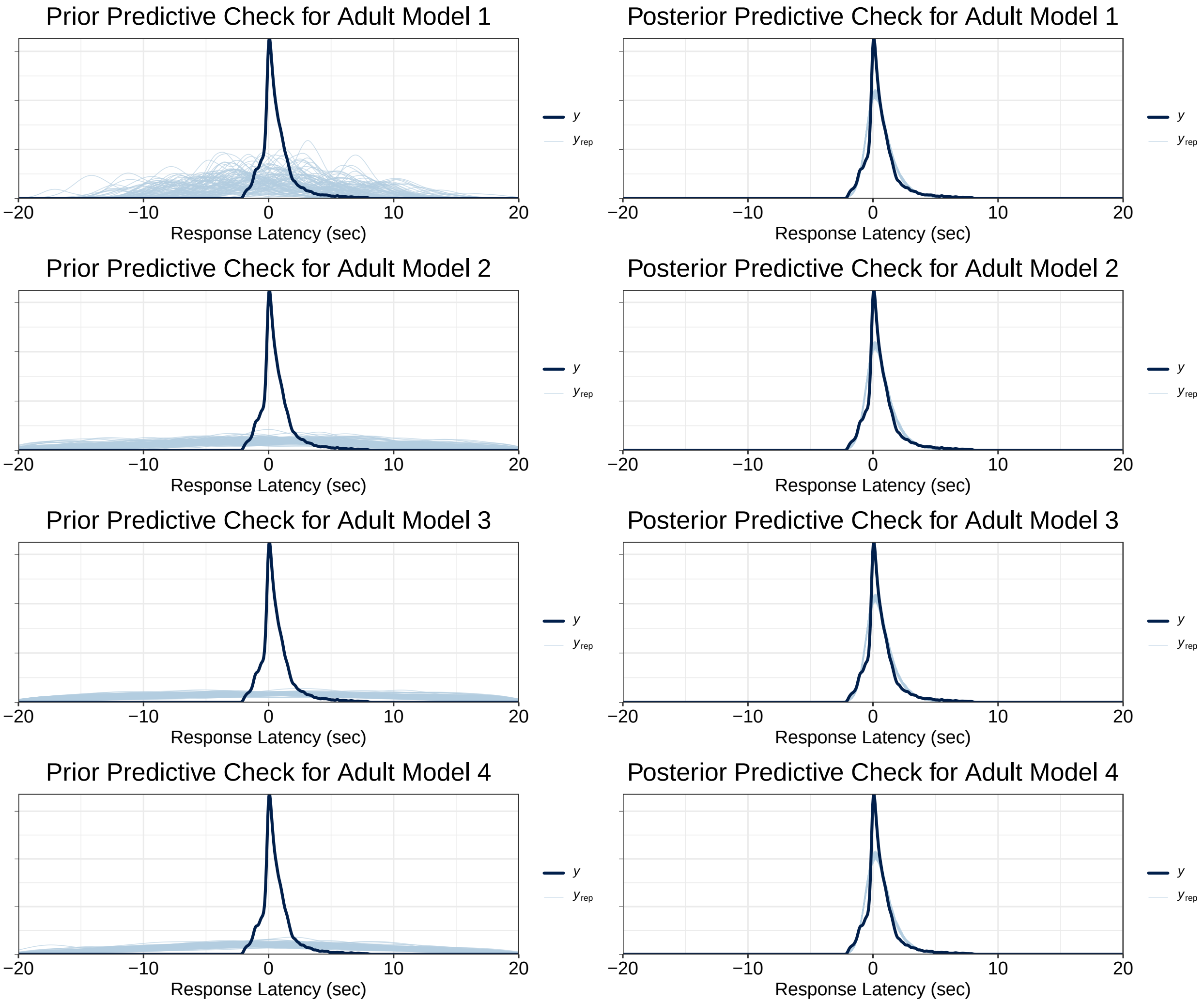

Figure S13: *Prior predictive checks (left column) and posterior predictive checks (right column) for adult latency in models 1-4.*

S.1.4.3 Prior-Posterior Update Plots

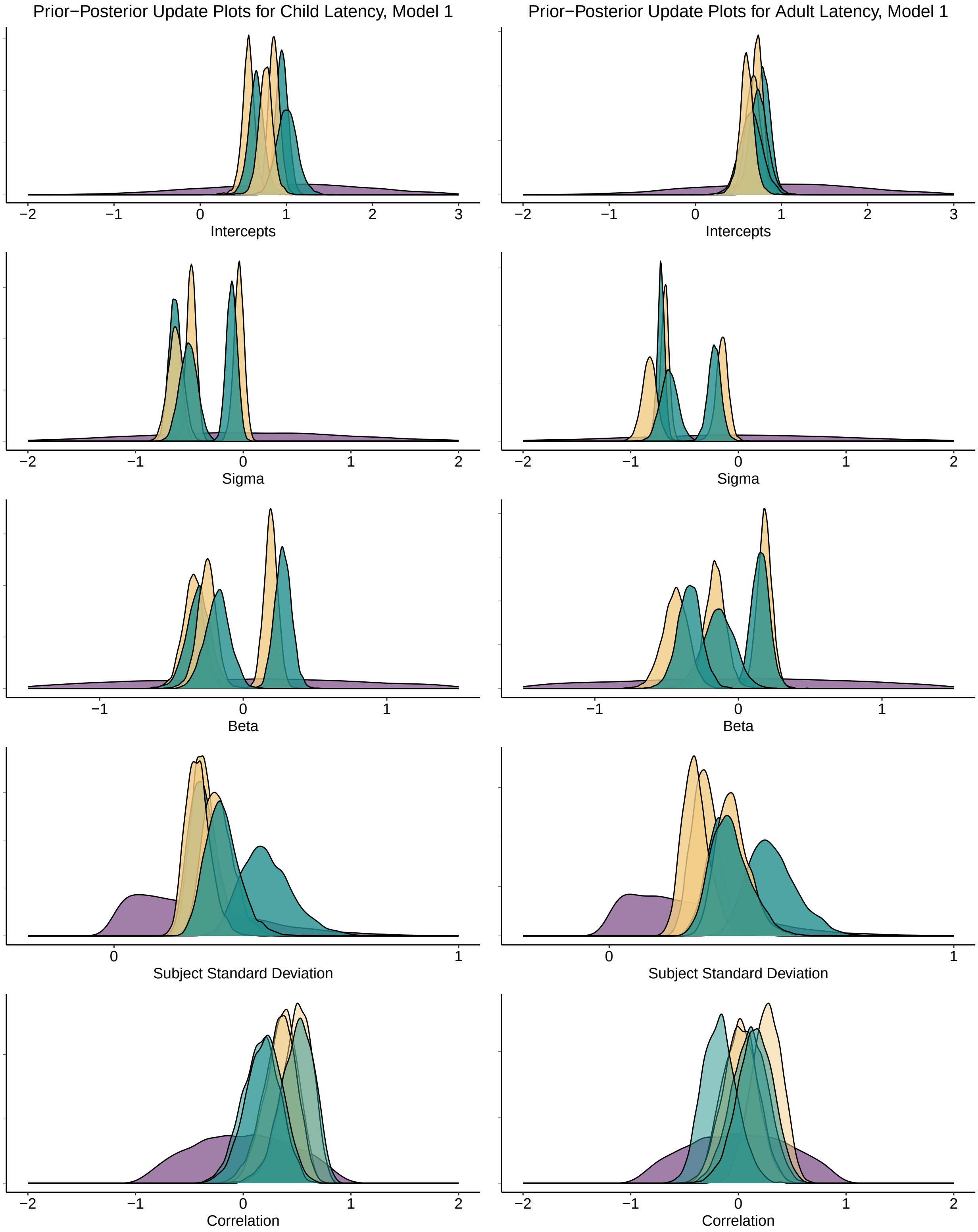

Figure S14: *Prior-posterior update checks for child and adult latency in Model 1. Purple density plots show the prior predictive density plot, turquoise density plots indicate posterior predictions for the typical development group, and yellow density plots denote the predicted estimates for the autism group.*

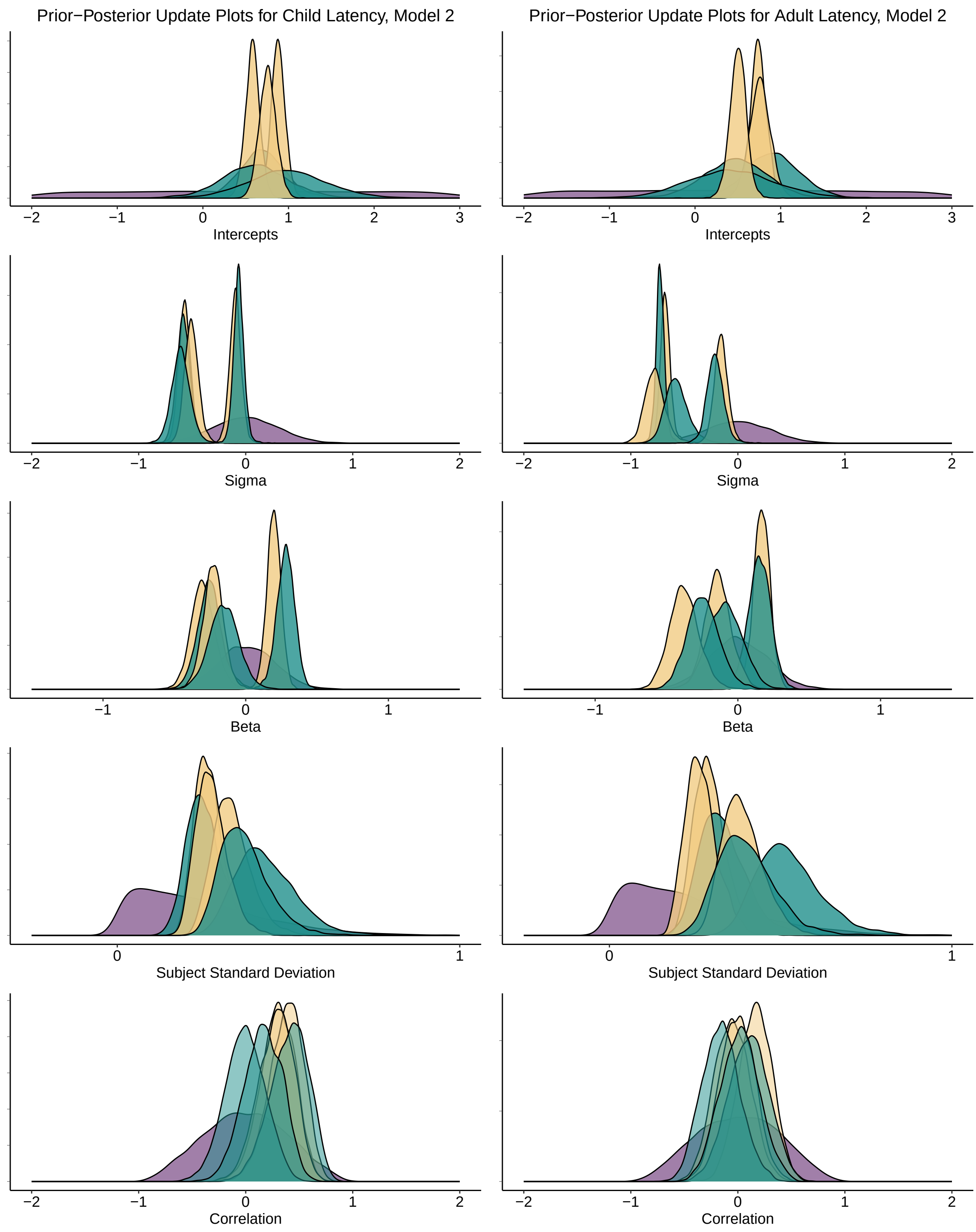

Figure S15: *Prior-posterior update checks for child and adult latency in Model 2. Purple density plots show the prior predictive density plot, turquoise density plots indicate posterior predictions for the typical development group, and yellow density plots denote the predicted estimates for the autism group.*

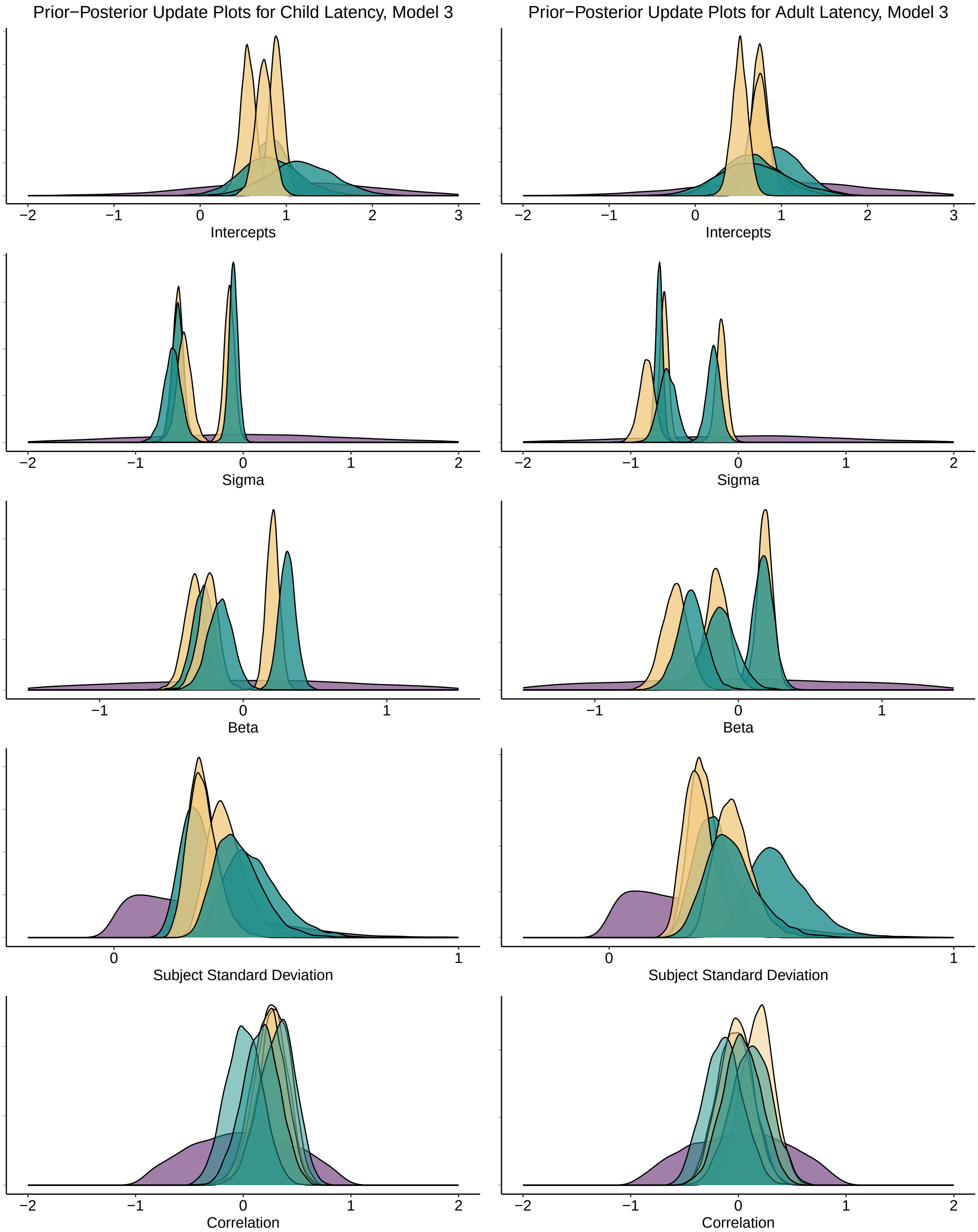

Figure S16: *Prior-posterior update checks for child and adult latency in Model 3. Purple density plots show the prior predictive density plot, turquoise density plots indicate posterior predictions for the typical development group, and yellow density plots denote the predicted estimates for the autism group.*

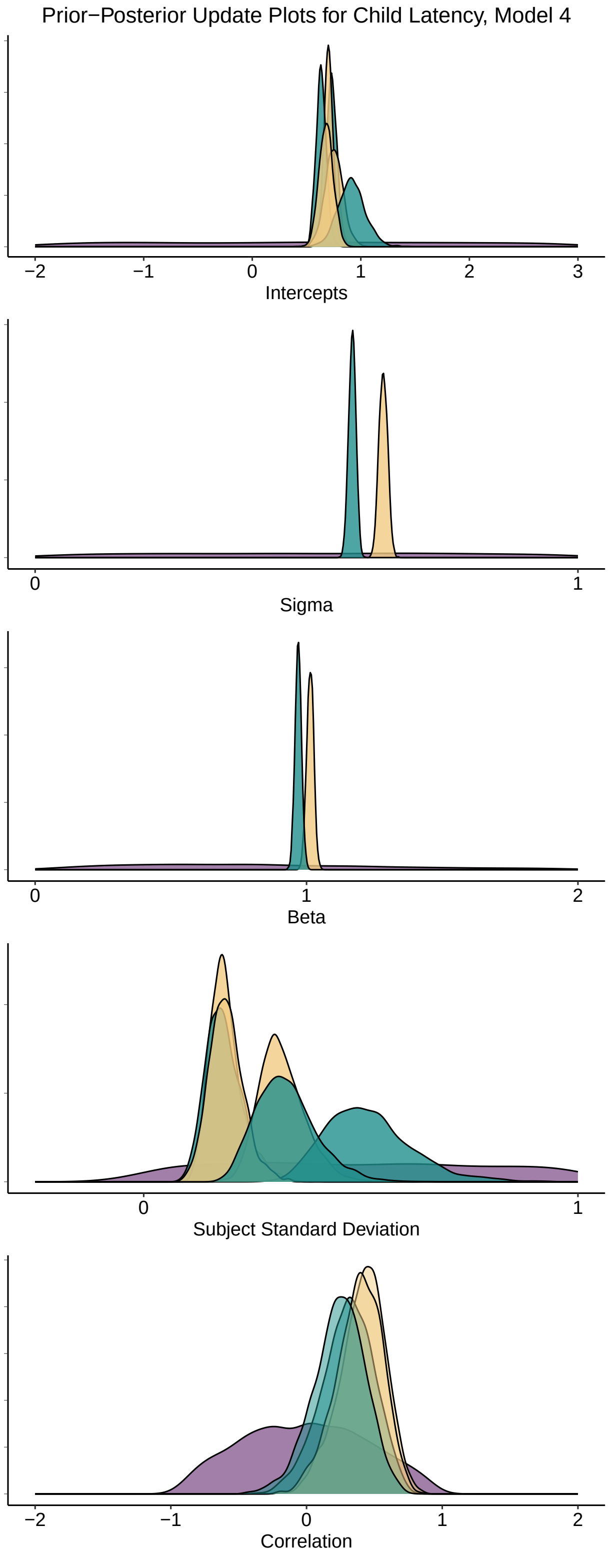

Figure S17: *Prior-posterior update checks for child and adult latency in the multivariate Model 4. Purple density plots show the prior predictive density plot, turquoise density plots indicate posterior predictions for the typical development group, and yellow density plots denote the predicted estimates for the autism group.*

S.1.5. Time Code Reliability

To assess the reliability of our transcription process, we compared two parallel transcriptions of the same audio recordings. Child speech segments were independently transcribed by two different transcribers (Transcriber 1 and Transcriber 2) across 104 conversation segments from our dataset, representing both structured task contexts (Matching Game) and open-ended conversations. Using a time-based comparison method with 50-millisecond intervals, we calculated Cohen's Kappa to measure agreement on speech presence/absence across the timeline of each recording, providing a robust measure of inter-transcriber reliability that accounts for chance agreement.

Our analysis revealed excellent agreement between transcribers in identifying child speech segments. The overall mean Cohen's Kappa was 0.88 (median = 0.89), indicating very high reliability between transcription methods. This level of agreement was consistent across both conversational contexts (mean $\kappa = 0.88$, 95% CI [0.74, 0.97]) and structured Matching Game tasks (mean $\kappa = 0.88$, 95% CI [0.76, 0.97]). The mean percentage agreement across all recordings was 94.7%, demonstrating that transcribers identified nearly identical patterns of child speech throughout the recordings.

Visual inspection of the alignment between transcription methods (**Figure S18**) confirms the strong temporal correspondence between transcribers. The histogram of Kappa values (**Figure S19**) shows that the majority of recordings achieved Kappa values above 0.85, with very few falling below 0.75, indicating consistently high reliability across the dataset. These results demonstrate that our transcription methodology produces highly reliable identification of child speech segments, providing a solid foundation for subsequent analyses of child language and communication patterns in our study.

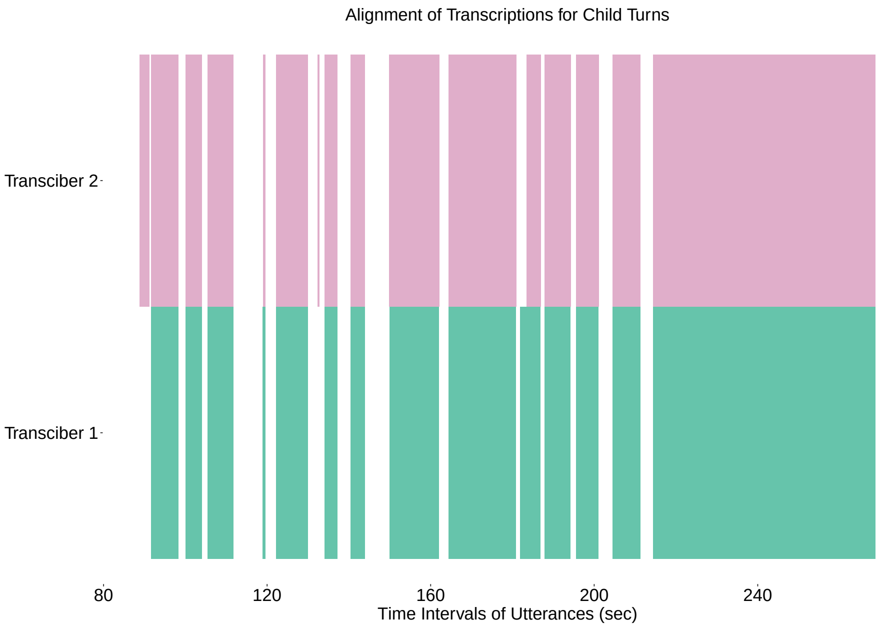

Figure S18: *Example of aligned transcriptions for child turns from a representative recording. The temporal alignment between Transcriber 1 (bottom, teal) and Transcriber 2 (top, pink) demonstrates the high degree of agreement in identifying child speech segments.*

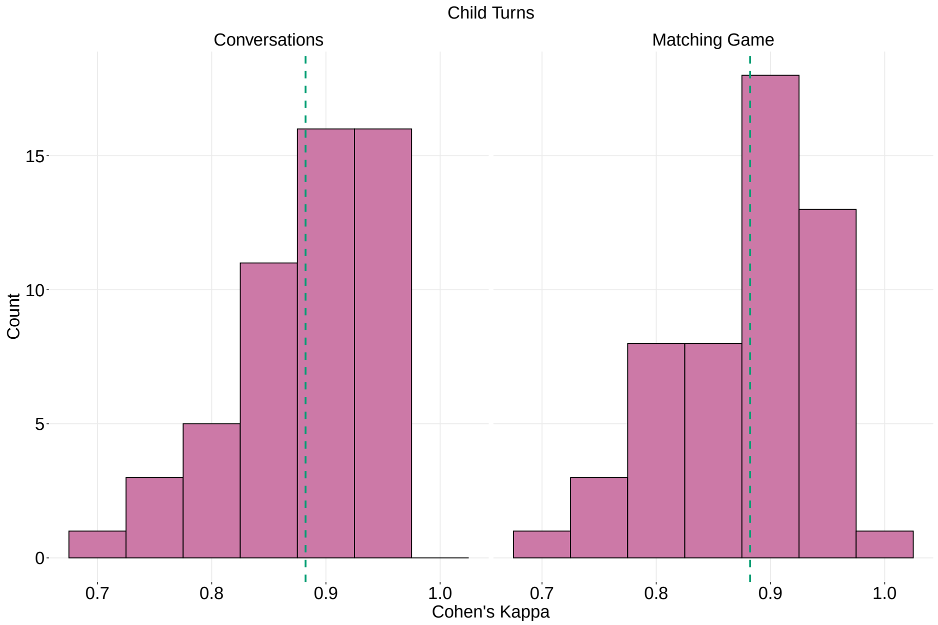

Figure S19: *Distribution of Cohen's Kappa values for child turns across different contexts. Left panel shows Kappa values for open-ended conversations (n=52); right panel shows Kappa values for structured Matching Game tasks (n=52). Vertical dashed lines indicate mean Kappa values for each context.*

S.1.6. Recurrence Analysis

Cross-Recurrence Quantification Analysis (CRQA) was employed to quantify non-linear dynamical coupling patterns in dyadic interactions across the three communicative tasks (Matching With Parent, Conversation With Parent, and Conversation With Experimenter) for both the Autism Group and Typical Development Group. CRQA offers a complementary perspective on bidirectional influences on behavioral patterns between interaction partners (R. Fusaroli, Konvalinka, & Wallot, 2014). For the CRQA computation, we used the following parameters: embedding dimension (m = 1), delay ($\tau = 1$), and radius ($\epsilon = 0.1 \times$ standard deviation of the normalized time series). These parameters were selected to capture the turn-taking dynamics in our interaction data while ensuring sufficient recurrence points for reliable analysis, and were applied consistently across all interaction samples to ensure comparability.

Figure S20 illustrates the distribution of Recurrence Rate (RR) across the three interaction contexts, demonstrating task-specific patterns of behavioral coordination. A Recurrence Rate (RR) of approximately 35% indicates that a third of all possible points in the interaction time series recur or synchronize, suggesting strong coordination between interaction partners. Table S14 presents a comprehensive set of CRQA metrics that characterize the temporal organization, predictability, and stability of interpersonal coordination in our sample. We report these exploratory analyses here to spur on further investigation in future research specifically designed to test dynamical hypotheses. We also used CRQA to examine directional coupling between child and adult behaviors across multiple time lags (-5 to +5) (see **Figure S21**). In conversations with experimenters, the autism group showed an increasing trend toward positive lags (suggesting adult-led dynamics), while the typical development group appears to exhibit a decline toward positive lags (potentially suggesting less adult-led structure in this context). The parent conversation condition presents a somewhat different picture, where the typical development group appears to demonstrate a possible trend toward adult-led dynamics,

while the autism group shows relatively stable patterns. In the structured matching task with parents, both groups show relatively modest variations across lags. These patterns suggest that interaction dynamics might involve different leadership patterns emerging depending on the social partner and task structure. Further research would need to explore the role of individual differences (e.g. in linguistic and socio-cognitive skills) for these dynamics and how they might impact the experience of the interaction.

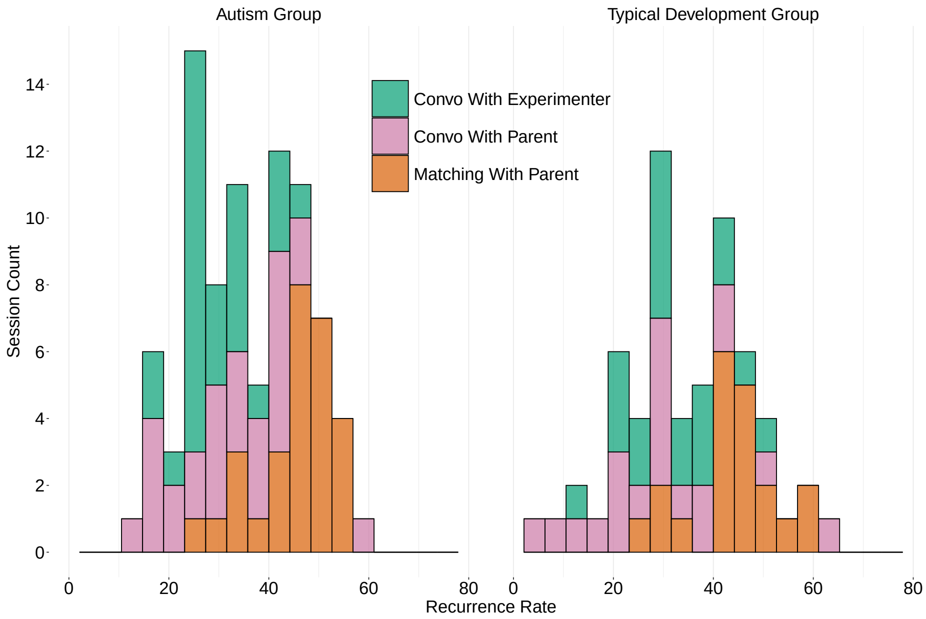

Figure S20: *Distribution of Recurrence Rate (RR) values across interaction tasks by diagnostic group. The histogram shows the frequency distribution of Recurrence Rate values for the Autism Group (left panel) and Typical Development Group (right panel), color-coded by interaction task: Conversation With Experimenter (green), Conversation With Parent (pink), and Matching With Parent (orange).*

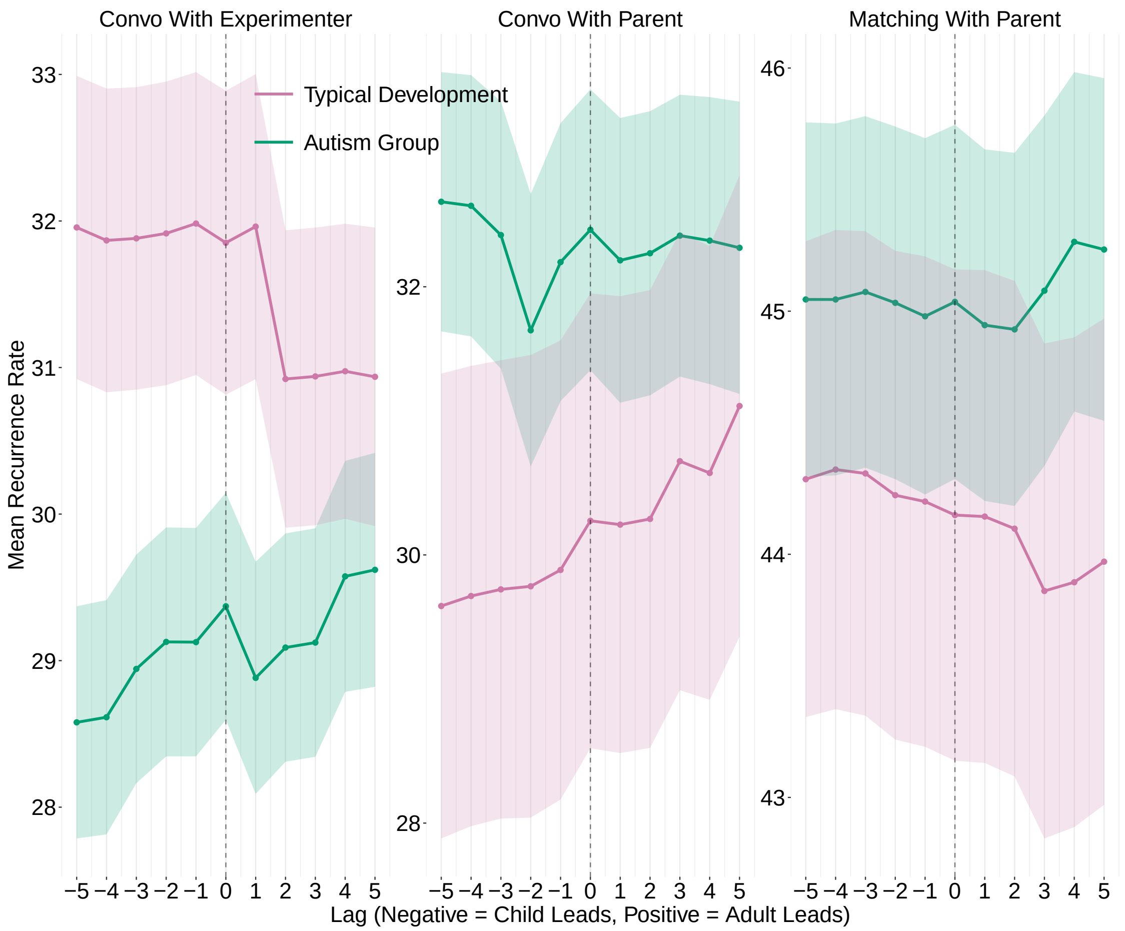

Figure S21: *Diagonal recurrence profiles revealing leader-follower dynamics across interaction contexts. Cross-recurrence quantification analysis across time lags (-5 to +5) examining directional coupling in dyadic interactions. Negative lags indicate child-led interactions, positive lags indicate adult-led interactions, and lag 0 represents synchronous patterns. Lines show group means for autism (green) and typical development (pink) groups. Shaded ribbons indicate ±0.5 standard errors.*

Table S14: *CRQA metrics quantify different aspects of recurrence patterns in dyadic interactions: RR (Recurrence Rate) represents the percentage of recurrent points in the recurrence plot, indicating overall similarity between behavioral patterns of interaction partners; DET (Determinism) measures the percentage of recurrent points forming diagonal lines, reflecting predictability and deterministic structure; LAM (Laminarity) indicates the percentage of recurrent points forming vertical lines, representing stability and resistance to change; L (Average Diagonal Line Length) quantifies the mean prediction time and duration of similar behavioral patterns between participants; TT (Trapping Time) represents the average length of vertical lines, indicating how long one participant's behavior remains in a particular state while the other changes; and ENTR (Entropy) reflects the complexity and diversity of diagonal line lengths, with higher values suggesting more complex interaction dynamics. Values are presented as means with 95% confidence intervals in brackets.*

	**Tasks**	**Autism Group**	**Typical Development**
**RR**	Matching With Parent	45.04 [29.93, 60.15]	44.16 [26.45, 61.87]
	Convo With Parent	32.43 [10.70, 54.15]	29.24 [1.85, 56.63]
	Convo With Experimenter	29.37 [13.30, 45.44]	31.85 [13.67, 50.03]
	Aggregate Estimate	35.61 [17.73, 53.49]	35.08 [13.52, 56.64]
**DET**	Matching With Parent	68.64 [51.01, 86.26]	68.20 [46.10, 90.30]
	Convo With Parent	53.49 [26.24, 80.75]	46.77 [7.96, 85.59]
	Convo With Experimenter	49.31 [27.03, 71.59]	51.67 [26.09, 77.26]
	Aggregate Estimate	57.15 [34.42, 79.87]	55.55 [25.83, 85.27]
**LAM**	Matching With Parent	77.71 [61.62, 93.79]	78.99 [65.39, 92.60]
	Convo With Parent	66.80 [41.30, 92.29]	59.24 [23.27, 95.20]
	Convo With Experimenter	59.59 [38.03, 81.16]	60.26 [34.28, 86.24]
	Aggregate Estimate	68.03 [46.63, 89.43]	66.16 [39.37, 92.96]
**L**	Matching With Parent	2.82 [2.27, 3.36]	2.83 [2.21, 3.46]
	Convo With Parent	2.50 [2.00, 3.00]	2.42 [1.68, 3.17]
	Convo With Experimenter	2.44 [2.02, 2.86]	2.48 [2.03, 2.93]
	Aggregate Estimate	2.58 [2.09, 3.08]	2.58 [1.96, 3.20]
**TT**	Matching With Parent	3.53 [2.41, 4.65]	3.53 [2.23, 4.83]
	Convo With Parent	3.07 [2.20, 3.95]	3.15 [0.81, 5.48]
	Convo With Experimenter	2.79 [1.94, 3.64]	2.85 [2.16, 3.54]
	Aggregate Estimate	3.13 [2.18, 4.09]	3.17 [1.58, 4.77]
**ENTR**	Matching With Parent	1.21 [0.72, 1.71]	1.23 [0.73, 1.72]
	Convo With Parent	0.89 [0.30, 1.47]	0.75 [-0.13, 1.62]
	Convo With Experimenter	0.85 [0.38, 1.32]	0.89 [0.39, 1.39]
	Aggregate Estimate	0.98 [0.46, 1.50]	0.95 [0.31, 1.60]

S.1.7. Research Assistants

S.1.7.1 Assignment of Research Assistants

Research assistant assignments across the four testing sessions (Sessions 1, 3, 5, and 7) are illustrated in **Figure S22**. We aimed to maintain consistent RA assignments for each participant across all sessions, but this was not always feasible due to scheduling constraints and RA availability. In the autism group (n=28), 20 participants (71.4%) had the same RA for all four sessions, while 8 participants (28.6%) had at least one session conducted by a different RA. Similarly, in the typical development group (n=20), 16 participants (80.0%) maintained the same RA throughout all sessions, while 4 participants (20.0%) experienced a change in RA assignment during the study. These RA changes were documented and considered in the analysis to ensure data integrity.

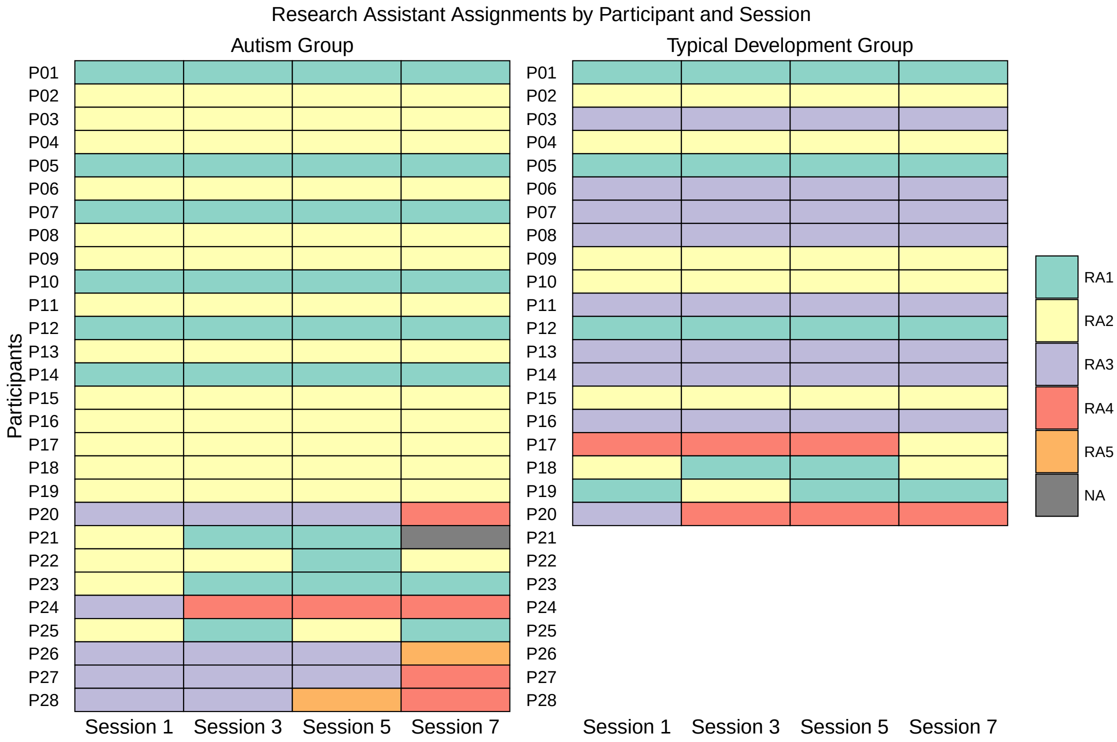

Figure S22: *Each row represents an individual participant, and each column represents a testing session (Sessions 1, 3, 5, and 7). Colors indicate different research assistants (RA1-RA5). Participants are arranged by RA consistency, with those having the same RA throughout all sessions appearing first, followed by those with RA changes during the study. The missing value (NA) for participant P21 in Session 7 indicates that this session was mistakenly conducted with the parent rather than a research assistant; data from this session therefore contributed to Conversation with Parent instead.*

S.1.7.2 Effects of Research Assistant

To address potential concerns about experimenter effects, we conducted a control analysis examining whether diagnostic differences in response latency were consistent across different research assistants. The diagnostic difference was consistent in both direction and rank ordering across all RAs: typically developing children showing longer response latencies than autistic children (0.23 to 0.45 seconds) (see **Figure S23**). Importantly, this effect was consistent across RAs: RAs who elicited slower responses from autistic children also elicited reliably slower responses from typically developing children, and there was no evidence of interactions between RAs and effect. This indicates that our primary findings reflect genuine

group differences rather than experimenter-specific artifacts.

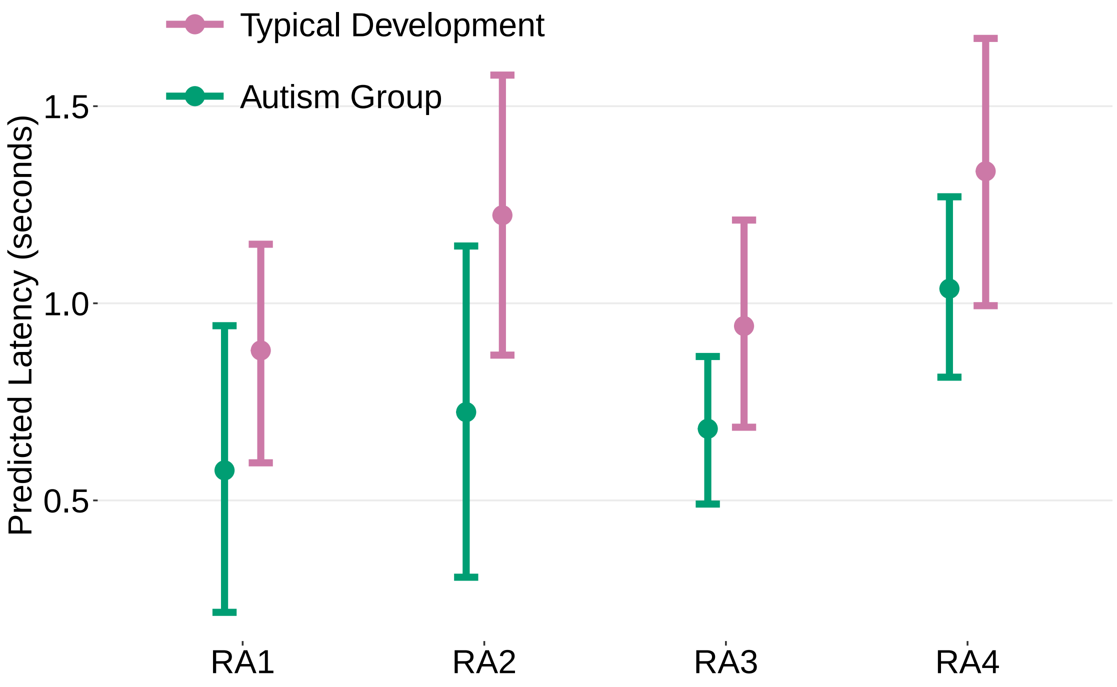

Figure S23: *Response latencies by diagnostic group across research assistants. Predicted response latencies (with 95% credible intervals represented by error bars) for conversations with unfamiliar experimenters, showing consistent diagnostic differences and rank ordering across four research assistants who conducted sufficient sessions for analysis, the fifth of which only conducted two sessions and only with children in the autism group.*

S.1.8. Recording Quality Assessment

All sessions were conducted via telephone using a dual-channel recording system in odd weeks and a single-channel recording system in even weeks. Technical issues were systematically documented for each session, including call connectivity problems, audio quality issues, and other disruptions that could potentially affect data collection or analysis (see **Table S15**).

Technical issues occurred in 28 of 336 total sessions (8.3%), distributed across both diagnostic groups and all testing sessions. The autism group experienced technical issues in 10 sessions (6.0% of 168 total sessions), while the typical development group encountered technical problems in 18 sessions (15.0% of 120 total sessions). The most common technical issue was temporary call disconnection followed by immediate reconnection, which occurred in 19 sessions (67.8% of all technical issues) and did not result in data loss or affect recording quality.

Recording quality was partially affected in 9 sessions (2.7% of all sessions), specifically due to audio breaking up or isolated muting events. These issues were distributed across both groups: 3 sessions in the autism group and 6 sessions in the typical development group. In all cases where recording quality was affected, the impact was deemed partial rather than complete, meaning that usable data could still be extracted from these sessions. The relatively minor technical issues had little effect on recording quality, and the timecodes showed good reliability (see **Section S.1.5**), suggesting that data collection was generally of high quality and reliable throughout the study.

Table S15: *Technical Issues by Session and Diagnostic Group. Technical issues were systematically documented across all testing sessions for both diagnostic groups. The "Issues" column shows the number of sessions with technical problems out of the total number of sessions conducted (e.g., 4/28 indicates 4 sessions with issues out of 28 total sessions). "Affected" indicates whether the technical issue impacted recording quality: "No" indicates the issue did not affect data quality (typically brief call disconnections with immediate reconnection), while "Partial" indicates some impact on audio quality that could potentially affect analysis (e.g., audio breakup, persistent muting events). Details specify the type and frequency of issues: Call dropped = temporary disconnection with reconnection; Breakup = partial audio quality degradation; Muting = isolated audio muting events. ASD = Autism Spectrum Disorder group; TD = Typical Development group.*

	**Session**	**Issues**	**Affected**	**Details**
**ASD**	1	4/28	Partial	Call dropped (2); Breakup (1); Muting (1)
	2	0/28	No	No issues
	3	1/28	No	Call dropped (1)
	4	0/28	No	No issues
	5	2/28	No	Call dropped (2)
	6	0/28	No	No issues
	7	3/28	Partial	Call dropped (2); Breakup (1)
**TD**	1	5/20	No	Call dropped (5)
	2	3/20	No	Call dropped (3)
	3	3/20	Partial	Call dropped (1); Breakup (1); Muting (1)
	4	1/20	No	Call dropped (1)
	5	2/20	Partial	Call dropped (1); Muting (1)
	6	1/20	No	Call dropped (1)
	7	3/20	Partial	Muting (3)
